# Assessment and Selection of Competing Models for Zero-Inflated Microbiome Data

**DOI:** 10.1371/journal.pone.0129606

**Published:** 2015-07-06

**Authors:** Lizhen Xu, Andrew D. Paterson, Williams Turpin, Wei Xu

**Affiliations:** 1 Dalla Lana School of Public Health, University of Toronto, ON, M5T 3M7, Canada; 2 Program in Genetics and Genome Biology, the Hospital for Sick Children Toronto, ON, M5G 0A4, Canada; 3 Division of Gastroenterology, Zane Cohen Centre for Digestive Diseases, Mount Sinai Hospital, Toronto, ON, M5T 3L9, Canada; 4 Department of Medicine, University of Toronto, ON, M5S 1A8, Canada; 5 Department of Biostatistics, Princess Margaret Hospital, 610 University Avenue, Toronto, ON, M5G 2M9, Canada; University of Rochester, UNITED STATES

## Abstract

Typical data in a microbiome study consist of the operational taxonomic unit (OTU) counts that have the characteristic of excess zeros, which are often ignored by investigators. In this paper, we compare the performance of different competing methods to model data with zero inflated features through extensive simulations and application to a microbiome study. These methods include standard parametric and non-parametric models, hurdle models, and zero inflated models. We examine varying degrees of zero inflation, with or without dispersion in the count component, as well as different magnitude and direction of the covariate effect on structural zeros and the count components. We focus on the assessment of type I error, power to detect the overall covariate effect, measures of model fit, and bias and effectiveness of parameter estimations. We also evaluate the abilities of model selection strategies using Akaike information criterion (AIC) or Vuong test to identify the correct model. The simulation studies show that hurdle and zero inflated models have well controlled type I errors, higher power, better goodness of fit measures, and are more accurate and efficient in the parameter estimation. Besides that, the hurdle models have similar goodness of fit and parameter estimation for the count component as their corresponding zero inflated models. However, the estimation and interpretation of the parameters for the zero components differs, and hurdle models are more stable when structural zeros are absent. We then discuss the model selection strategy for zero inflated data and implement it in a gut microbiome study of > 400 independent subjects.

## Introduction

The human microbiome plays an important role in human disease and health. The advent of next-generation sequencing (NGS) technology enables researchers to quantify the organisms present in the community using direct DNA sequencing without the need for laborious cultivation [1, 2]. The process starts with the collection of human associated samples and successful extraction of the bacterial DNA. The hypervariable regions of bacterial 16S rRNA gene are then PCR-amplified and sequenced. The processed sequences are clustered into operational taxonomic units (OTUs) at a certain similarity level in a taxonomic independent way. Typical data in a microbiome study consist of the OTU counts that have the complexity of non-negative, over-dispersed, and having a large number of zeros. The zero inflation of the microbiota abundance is due to the fact that the OTUs are subject dependent, i.e. their composition is unique in each subject. As a result, only a few major bacterial taxa of the microbiota are shared across samples and the rest are detected only in a small percentage of the samples. The zero counts in the sample could be due to either simply being absent (structural zeros), or present with low frequency but not observed because of sampling variation (sampling zeros).

It is often of interest to determine whether the abundance of one or more OTUs is associated with some environmental or genetic factors. For example, several studies have revealed the relationships between microbial composition and obesity [3, 4] and type 2 diabetes [5, 6]. So far there is no standard statistical method to evaluate such relationships. Most of the current methods are based on classical linear regression or logistic regression models [7–12]. To adjust for variation in the number of total sequence reads across samples, relative abundance is usually used as the outcomes in the model. It is well known that classical linear models using either non-transformed or logarithmic transformed counts are inappropriate for zero inflated count data due to the violation of normality and constant variance assumptions [13]. The normality and homogeneity of variance assumptions are not relevant for relative abundance either. For example, relative abundances are bounded by zero and one and the variance is often mean dependent. Furthermore, no data transformation can satisfy the assumptions if excess zeros are present. Logistic regression treating all the zero counts as non-events is commonly used to handle zero inflated OTU count data. However it will result in the loss of valuable information and lower power to detect a covariate effect. Although non-parametric models such as Wilcoxon rank sum (WRS) test are used as alternative ways to avoid the normality assumption [14–16], they have the limitation of being unable to incorporate covariates, as well as the potential loss of power because of the large number of ties caused by many zeros [17]. Generalized linear models such as Poisson or negative binomial (NB) model can be applied on sequence counts and the logarithm of total sequence reads can be set as an offset. However, they cannot account for the excess zeros either, because a basic requirement of these models is that the proportion of zeros must be necessarily linked to the distribution of the positive values [18].

One way to deal with many zeros is to use a zero inflated (ZI) model [19], which is essentially a mixture of a Poisson or NB model with a point mass at zero to allow for the inclusion of structural zeros. Another approach is to use a hurdle model [20], also called a two-part model, with the first part being a binomial probability model to determine whether a zero or non-zero outcome occurs; and the second being count data truncated-at-zero to analyze the positive counts. Unlike ZI models, hurdle models do not make the distinction between structural and sampling zeros and handle them identically. Both hurdle and ZI models have been used in a variety of areas such as psychology [13], ecology [18, 21], manufacturing [19], and public health [22–24]. However, they are rarely used in human microbiome studies.

It is desired to have a comprehensive comparison of different model performance for zero inflated data, focusing on the pattern of superiority using hurdle/ZI models and limitations of one part models. Some simulation studies in the literature compared different model performance for data with excess zeros [25–27]. However, the comparisons in these studies are limited. For example, Min and Agresti [25] focused on comparing the parameter estimations of Poisson hurdle (PH) with zero inflated Poisson (ZIP); Miller [26] compared the goodness of fit for Poisson, PH and ZIP; and Desjardins [27] compared the model performance of zero inflated negative binomial (ZINB) with negative binomial hurdle (NBH). In addition, although Desjardins [27] evaluated type I error rate separately for the structural zero and count component, no evaluations have been conducted on the overall Type I error rate and statistical power in these studies.

In this paper, we conduct a comprehensive comparison of the performance of different possible competing models through simulations for zero inflated count data from different perspectives such as type I error, power of the test, the precision and efficiency of parameter estimations of the covariate effect on both the counts and the (structural) zeros, the goodness of fit, and the relative bias of prediction for zeros. Two sets of simulations are conducted under the ZIP and ZINB distributions. The model fit is based on a regression framework, with one binary covariate in the model for illustration. We first briefly outline the existing approaches to model count data with excess zeros (Section Summary of competing methods used for model comparison), we then discuss how to select the most appropriate models for a specific study (Section Model selection). The simulation settings are introduced in Section Simulation settings. Results of model fitting are compared for type I error and the power to detect a significant effect (Section Hypothesis testing of the covariate effect). The performances of parametric approaches on the accuracy, efficiency and goodness of fit of statistical inference are also inspected (Section Estimation of the covariate effects and AIC values). Additionally, we evaluate the abilities of model selection strategies using Akaike information criterion (AIC) or Vuong test [28] to identify the correct model (Section Evaluation of model selection procedure). We then apply different methods to a gut microbiota study and discuss the selection of appropriate models for three bacteria abundance data at the genus level of phylogenetic bacterial classification (Section Application to human microbiome study).

## Methodology

### Summary of competing methods used for model comparison

We classify the possible competing methods into three categories according to how the excess zeros are treated: one-part, zero inflated and hurdle (or two-part) models.

#### One part models

The one-part models refer to the models that ignore the existence of the excess zeros and model the data using either standard distributions or based on ranks. They include Poisson model, NB model, ordinary least squares on logarithmic transformed data (LOLS), and the non-parametric WRS test.

Both Poisson and NB model are classical generalized linear models (GLM) for count data, with NB addressing over-dispersion in the data. In practice, LOLS is also commonly used for abundance count data in order to transform it to be more normally distributed [2, 29]. To deal with zero observations, a constant *a* should be first added to the original data before taking the log transformation. In this paper, we set *a* = 1. When normality assumption is still violated after transformation, the Wilcoxon rank-based approaches are usually recommended.

#### Zero inflated models

The zero inflated models include ZIP and ZINB and assume that for each observation, there are two possible data generation processes with the result of a Bernoulli trial determining which process is used. The first process generates only zero counts (structural zeros, denoted as {0} hereafter.), while the second generates counts from either a Poisson or NB model. If the probability of structural zeros is denoted as *ϕ*, the probability function of *Y* can be written compactly as: *f*(*y*) = *ϕd*(*y*) + (1 − *ϕ*)*g*(*y*), where *d*(*y*) = 1 − *min*(*y*,1) and *g*(*y*) is a regular count data probability function such as the Poisson or the NB probability function. To examine the effects of risk factors on the response variable, Lambert [19] proposed the ZIP regression model to allow both *ϕ* and the Poisson mean *λ* to depend on some covariates through canonical link GLMs as $\text{log}(\lambda_{i})={\text{γ}}_{\text{0}}+{\text{X}}_{\text{i}}^{\text{T}}\text{γ}$ and $\text{logit}(\phi_{i})=\text{log}(\phi_{i}/(1-\phi_{i}))=\beta_{0}+{\text{W}}_{\text{i}}^{\text{T}}\text{β}$ for the *i* ^*th*^ subject, where ***X***_***i***_ and ***W***_***i***_ denote the vector of covariates for *λ*_*i*_ and *ϕ*_*i*_, respectively. Similarly, the ZINB regression model allows both *ϕ* and the mean of the count component to depend on some covariates through a binomial logistic regression and a NB log linear regression, respectively.

Notice that a covariate can have effects on both structural zeros and the count component. A covariate is said to have “consonant effects” if higher values are associated with a lower proportion of structural zeros and higher count component means, or vice versa, i.e., if its corresponding regression coefficients *β* and *γ* have opposite signs [17]. It is “consonant” because it works in the same direction on the two ZI parts in increasing or decreasing the outcome overall mean. Covariates with this feature are commonly observed in health studies. When the signs of *β* and *γ* are the same, the covariate is said to have “dissonant effects” as it works in an opposite direction on the two ZI parts in affecting the overall mean. An example of this case is an antibiotic treatment that may be effective in reducing the risk of carrying some specific bacteria, but may result in the growth of these bacteria once they survive due to antibiotic resistance. If a covariate only has an effect on the count component, we follow Lachenbruch′s terminology [17] and say that it has “neutral effects” on the outcome.

#### Hurdle models

The hurdle models refer to those that divide the modeling stage into two parts to correct for excess zeros. The first part determines whether the response outcome is positive via a binary model for the dichotomous event of having zero or positive values and logistic regression is usually used to allow for the investigation of the effects (denoted as $\tilde{\beta }$) of covariates **W** on the probability of an observation being zero (denoted as *π*_0_). Then conditioning on it being positive, the second stage models the level of the outcome which is a truncated-at-zero count outcome. Typical choices for the truncated-at-zero count model are truncated Poisson for PH model [20], or truncated negative binomial model for NBH model. Log-linear models are then used to investigate the effects (denoted as $\tilde{\gamma }$) of covariates **X** on the mean (denoted as *λ*) of the un-truncated Poisson or NB distribution. In practice, the 2P-LOLS model [30] which assumes that the positive data follow a log-normal distribution, is also used to model the count data especially when the data are highly skewed [31, 32]. If no parametric assumption is made on the distribution of the positive counts, the non-parametric two-part WRS test (2P-WRS) can be used [33, 34].

Notice that if *ϕ* is constant across the samples, the PH (NBH) can be considered as a re-parameterization of ZIP (ZINB) although in general this is not the case. In fact, when covariates are included in the regression model of the zero part, their effects ($\tilde{\beta }$) on *π*_0_ in a hurdle model and effects (***β***) on *ϕ* in a ZI model are not equivalent as they refer to entirely different parameters (i.e., $\tilde{\beta }$ refer to the covariate effects on the log-odds of a zero response, while ***β*** refer to the covariate effects on the log-odds of structural zeros.), However, in our simulation settings with a single binary predictor for both the count and zero components, there is an equivalence relationship between β in PH and ***β*** in ZIP through

$$\begin{array}{ccc} \exp({\tilde{\beta }}_{0}) & = & \frac{\left(1+\exp(\beta_{0})\right)}{\left[1-\exp(-\exp\left(\gamma_{0}\right))\right]}-1\\ \exp(\tilde{\beta_{0}}+{\tilde{\beta }}_{1}) & = & \frac{\left[1+\exp(\beta_{0}+\beta_{1})\right]}{\left[1-\exp(-\exp(\gamma_{0}+\gamma_{1}))\right]}-1. \end{array}$$ (1)

For $\tilde{\beta }$ in NBH and ***β*** in ZINB, the equivalence relationship is through

$$\begin{array}{ccc} \exp({\tilde{\beta }}_{0}) & = & \frac{\left(1+\exp(\beta_{0})\right)}{\left[1-{\left(\frac{1}{1+\kappa \exp(\gamma_{0})}\right)}^{\kappa^{-1}}\right]}-1\\ \exp({\tilde{\beta }}_{0}+{\tilde{\beta }}_{1}) & = & \frac{\left(1+\exp(\beta_{0}+\beta_{1})\right)}{\left[1-{\left(\frac{1}{1+\kappa \exp(\gamma_{0}+\gamma_{1})}\right)}^{\kappa^{-1}}\right]}-1, \end{array}$$ (2)

where *κ* is the over-dispersion parameter for the count component in ZINB model. The count part of a PH (NBH) has the same parameters as the count component of the corresponding ZIP (ZINB) model.

### Model selection

A critical question in data analysis is how to choose the appropriate models for a specific study. Model selection should be based on quantitative assessment, qualitative information (e.g. clinical relevance of parameter estimates), and the study purpose. Several criteria can be used to compare and select among considered models.

To see whether the dispersion parameter is necessary, likelihood ratio and/or score tests can be used to compare nested models: Poisson vs. NB; ZIP vs. ZINB; and PH vs. NBH. To test whether excess zeros exist in the data, we can compare ZIP (or PH) vs. Poisson, ZINB (or NBH) vs. NB. Notice that likelihood ratio or score tests are not applicable since the models compared are not nested. One common way to test non-nested models is to use Vuong test [28]. The information criterion such as AIC or Bayesian information criterion (BIC) provides another way to compare both non-nested and nested models. The AIC is computed using the formula *AIC* = −2*log*(*L*) + 2*q*, where *L* is the likelihood and *q* is the number of parameters in the model. In general, the best fitting model has the lowest AIC value.

It should be noted that for LOLS and 2P-LOLS, continuous distributions are being fitted to discrete data, but the log-likelihood of discrete and continuous distributions are not comparable. To compare their AICs with the models based on discrete distributions, we discretized the Gaussian distribution for AIC calculations [29]. For example, the log-likelihood of LOLS is calculated using:

$$\begin{array}{ccc} & & l(\gamma_{0},\gamma ,\sigma^{2};y)\\ & & \;=\sum_{i=1}^{N}\;log\{\Phi [\frac{log\left(y_{i}+a+0.5\right)-\hat{\gamma_{0}}-X_{i}^{T}\hat{\gamma }}{\hat{\sigma }}]-\Phi [\frac{log\left(y_{i}+a-0.5\right)-\hat{\gamma_{0}}-X_{i}^{T}\hat{\gamma }}{\hat{\sigma }}]\}, \end{array}$$

where Φ is the cumulative distribution function of the standard normal distribution. The calculation of AIC for 2P-LOLS can be done in a similar way.

### Simulation settings

The simulation studies focus on the scenarios that structural zeros are present in the data, and there is only one binary covariate in both the structural zero part and the count component. The binary covariate *x*_*i*_ is defined as an indicator of the exposed group and the probability of an individual coming from the exposed group is set as 50%. 1000 subjects are generated in each simulation.

Data are simulated under ZIP and ZINB distribution. To generate the simulation data, for each subject *i*, first we simulate *Z*_*i*_ from a binomial distribution where logit(*p*(*Z*_*i*_ = 1)) = logit(*ϕ*_*i*_) = *β*_0_ + *β*_1_ *x*_*i*_. Then, if *Z*_*i*_ = 1, we set the outcome *Y*_*i*_ to be zero; and if *Z*_*i*_ = 0, we simulate *Y*_*i*_ from either a Poisson distribution with *Y*_*i*_ ∼ Poisson(*exp*(*γ*_0_ + *γ*_1_ *x*_*i*_)) for ZIP distributed data or a NB distribution with *Y*_*i*_ ∼ *NB*(*exp*(*γ*_0_ + *γ*_1_ *x*_*i*_),*κ*) for ZINB distributed data.

**Fig 1:**
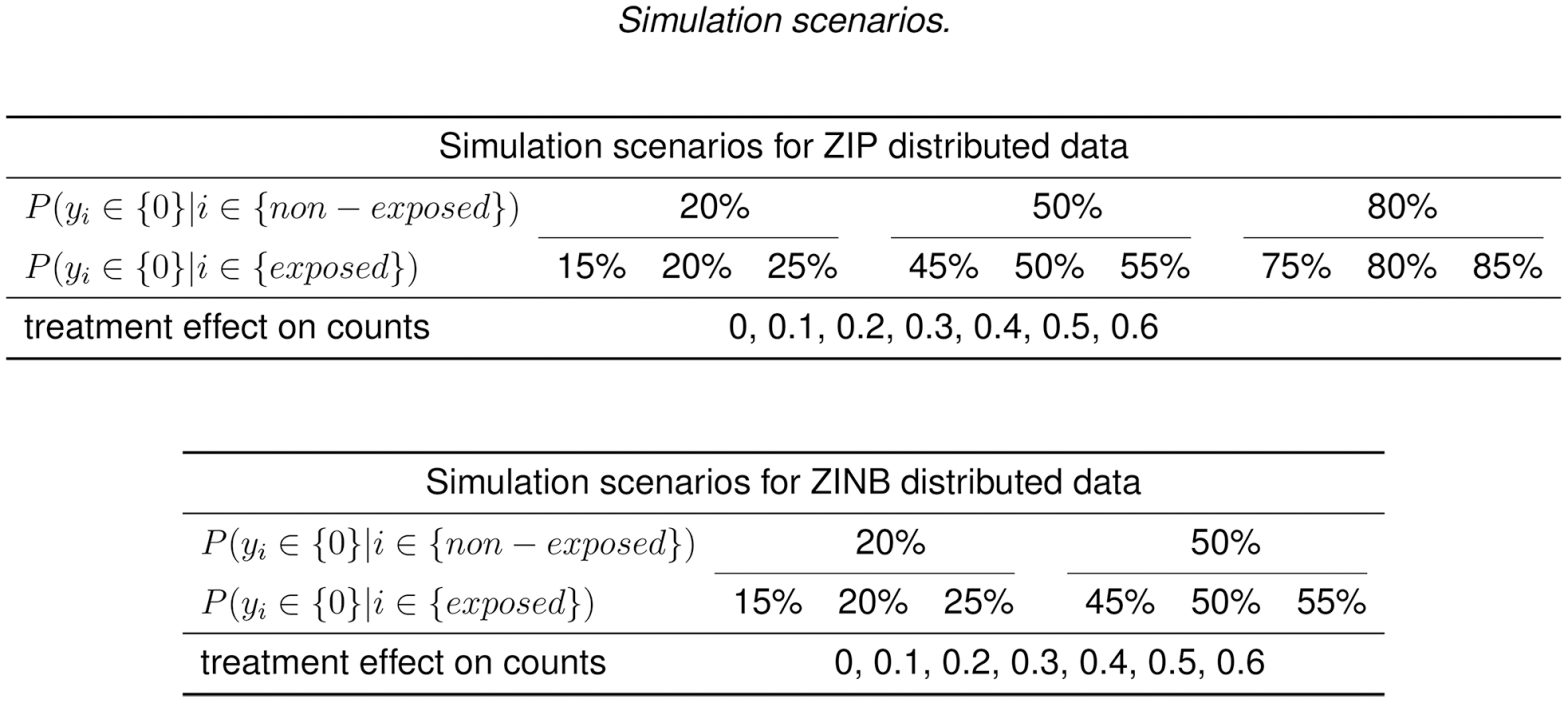
The simulation scenario. *γ*_0_ = 1 for all simulation scenarios. The over-dispersion parameter *κ* is set to be 1 for all ZINB simulation scenarios. *β*_0_ reflects the log odds of zero inflation in the unexposed group, and is equal to {−1.386, 0, 1.386} for the {20%, 50%, 80%} of zero inflation in this group. *β*_1_ reflects the change in log odds of zero inflation when changing from unexposed to exposed group. The corresponding values of *β*_1_ of {−5%, 0, +5%} changing in the zero inflation are {−0.349, 0, 0.287}, {0.201, 0, 0.201}, and {−0.287, 0, 0.349} for 20%, 50% and 80% of the zero inflations in the unexposed group, repsectively.

We consider a factorial design in which the factors are the proportion of zero inflation in the unexposed group, the exposure effect on the count component, as well as on the structural zeros (Fig 1).

**Fig 2:**
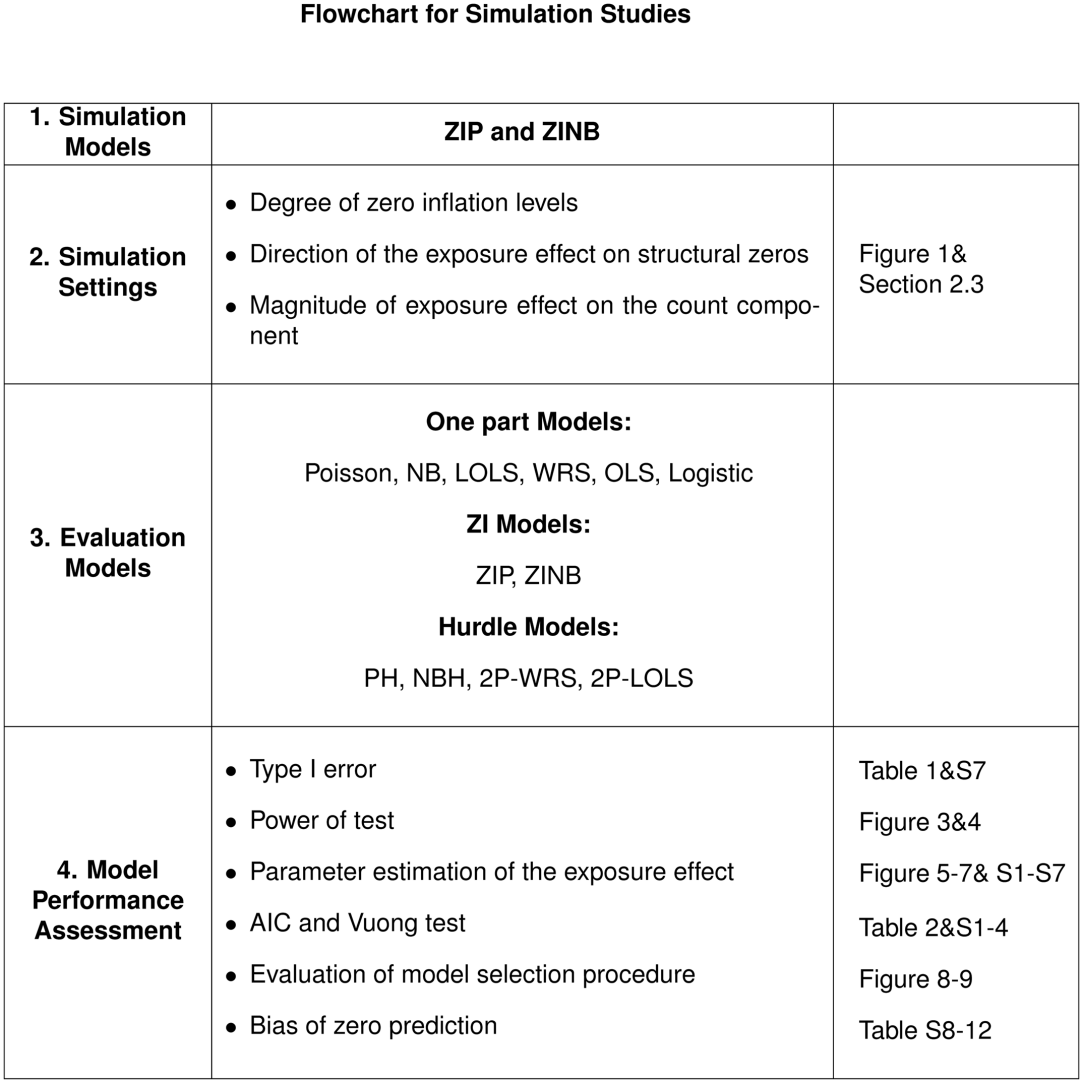
The flowchart for simulation studies.

We generate 1,000 datasets for each simulation scenario and fit the data using different methods such as LOLS, Poisson, NB, ZIP, ZINB, PH, NBH, and 2P-LOLS assuming that the exposed/unexposed group indicator *X* is the only predictor in the models. For the hurdle/ ZI models, *X* is the predictor for both the probability of zeros/structural zeros and the count component. For comparison, we also apply the non-parametric WRS, 2P-WRS, OLS and logistic regression in the hypothesis testing in the significance of the exposure effect. The flowchart of the simulation studies is shown in Fig 2.

## Results

We compare the model fitting results from different perspectives. Simulations show that, in many situations, hurdle count models (PH and NBH) produce identical fitting results as their corresponding ZI models. If the results of PH is the same as ZIP, then PH/ZIP is used to present the results for both PH and ZIP. Similarly, NBH/ZINB is used to present the results of NBH and ZINB when their results are the same.

### Hypothesis testing of the covariate effect

To test the significance of covariate effect, we perform the hypothesis test on *H*_0_: *γ*_1_ = 0 vs. *H*_*A*_: *γ*_1_ ≠ 0 using Wald test statistics for the one part parametric models; for the hurdle/ZI models, likelihood ratio test statistics is used to test *H*_0_: *β*_1_ = 0 (${\tilde{\beta }}_{1}=0$ for hurdle models); *γ*_1_ = 0 vs. *H*_*A*_: not both are equal to 0. For the WRS test, the significance test for the covariate effect is just equivalent to testing whether there is a significant location shift between the exposed and unexposed groups. For the 2P-WRS method, we use the test statistic *χ* ^2^ = *Z* ^2^ + *U* ^2^ [33], where *Z* is the test statistic of the logistic regression for the first part of the model and *U* being the rank-sum statistic based on the non-zero data. This test statistic follows a *χ* ^2^ distribution with two degrees of freedom.

#### The overall type I error rates

The type I error rates are estimated using the proportion of data sets for which the null hypothesis was falsely rejected, i.e., the percentages of detecting significant overall covariate effect for 10,000 replications when the true value of *β*_1_ and *γ*_1_ are all equal to zero. Table 1 shows the estimated type I error rates at significance levels *α* = {0.05,0.1} using different methods for the simulated data sets. Results show that Poisson regression has a substantially inflated type I error for both ZIP and ZINB distributed data, and so does PH/ZIP for ZINB distributed data. On the other hand, NB method yields fewer false positive than would be expected by chance, and the deflation is more obvious when the proportion of structural zeros is 50% or more. The type I error rates of other methods are appropriate.

**Table 1:** The type I error rate estimations.

	ZIP distributed data						ZINB distributed data			
*ϕ*_*c*_	20%		50%		80%		20%		50%	
*α*	.05	.10	.05	.10	.05	.10	.05	.10	.05	.10
LOLS	.052	.101	.051	.105	.054	.104	.052	.101	.050	.099
Poisson	**.117**	**.193**	**.205**	**.287**	**.273**	**.361**	**.345**	**.422**	**.394**	**.471**
NB	.045	.090	.027	.060	.028	.059	.040	.085	.031	.069
WRS	.050	.102	.052	.105	.054	.106	.053	.103	.050	.099
2P-LOLS	.050	.103	.053	.110	.060	.113	.054	.104	.051	.100
PH/ZIP	.053	.104	.053	.104	.054	.104	**.219**	**.306**	**.224**	**.309**
NBH/ZINB	.051	.103	.049	.098	.051	.100	.051	.098	.057	.112
2P-WRS	.047	.099	.047	.101	.044	.094	.053	.103	.049	.098

Estimates are based on 10,000 replicated samples. *ϕ*_*c*_ is the probability of *y* coming from structural zeros for the unexposed group. *α* is the significant level of test. A bold value represents inflated type I error.

#### Power of test

**Fig 3:**
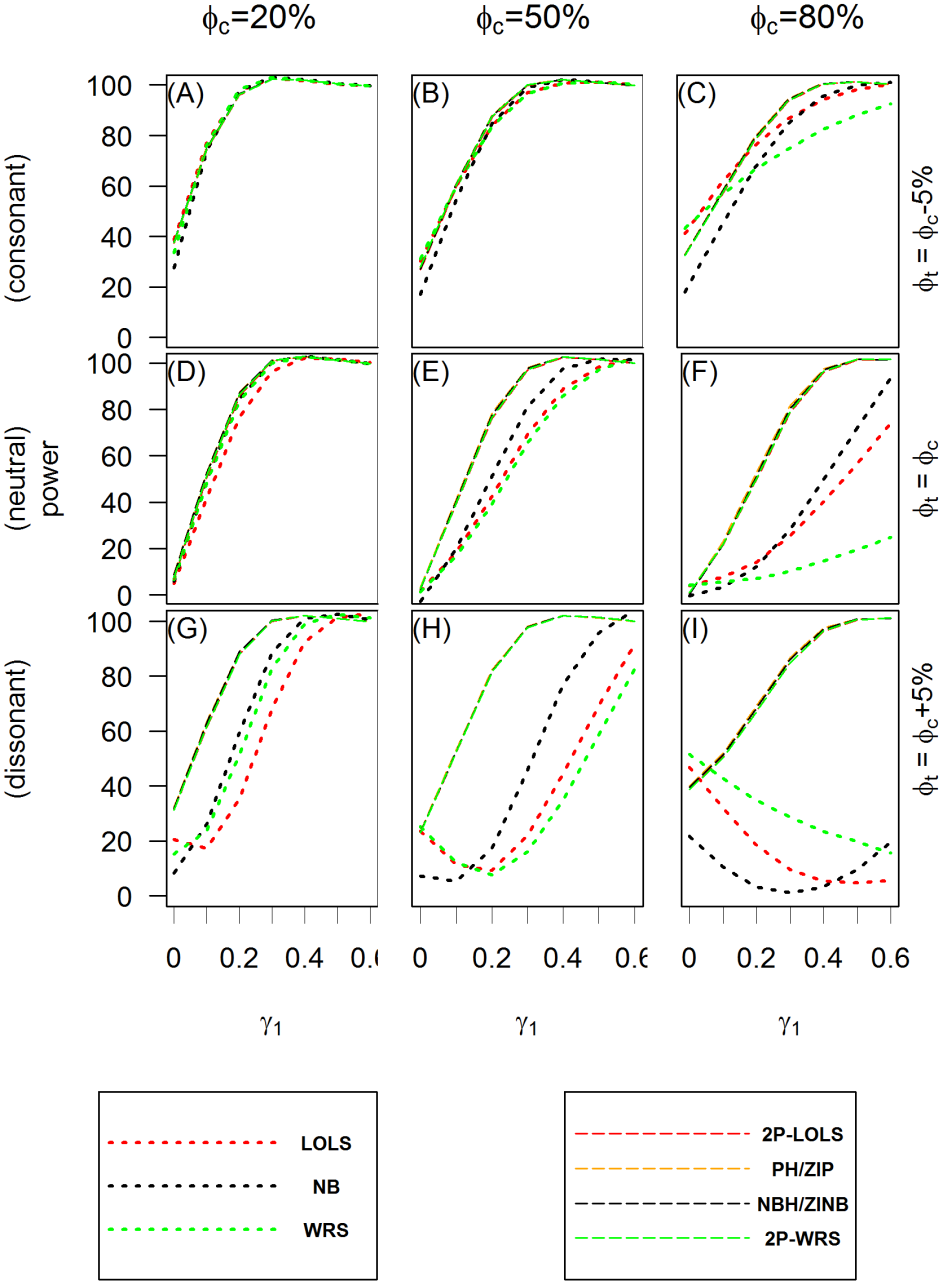
The power of test for ZIP simulated data. The *X* axis is the value of the covariate effect on the count data *γ*_1_ and the *Y* axis is the power of test when the level of significance is 0.05. Three different cases of covariate effect, i.e., the consonant (*ϕ*_*t*_ = *ϕ*_*c*_ − 5%), neutral (*ϕ*_*t*_ = *ϕ*_*c*_) and dissonant (*ϕ*_*t*_ = *ϕ*_*c*_ + 5%) effect, are presented in panels **(A)**, **(B)** and **(C)**; **(D)**, **(E)** and **(F)**; and **(G)**, **(H)** and **(I)**, respectively. Each column reflects different proportion of zero inflation in the unexposed group: 20% in **(A)**, **(D)** and **(G)**; 50% in **(B)**, **(E)** and **(H)**; and 80% in **(C)**, **(F)** and **(I)** from the first to the third column.

**Fig 4:**
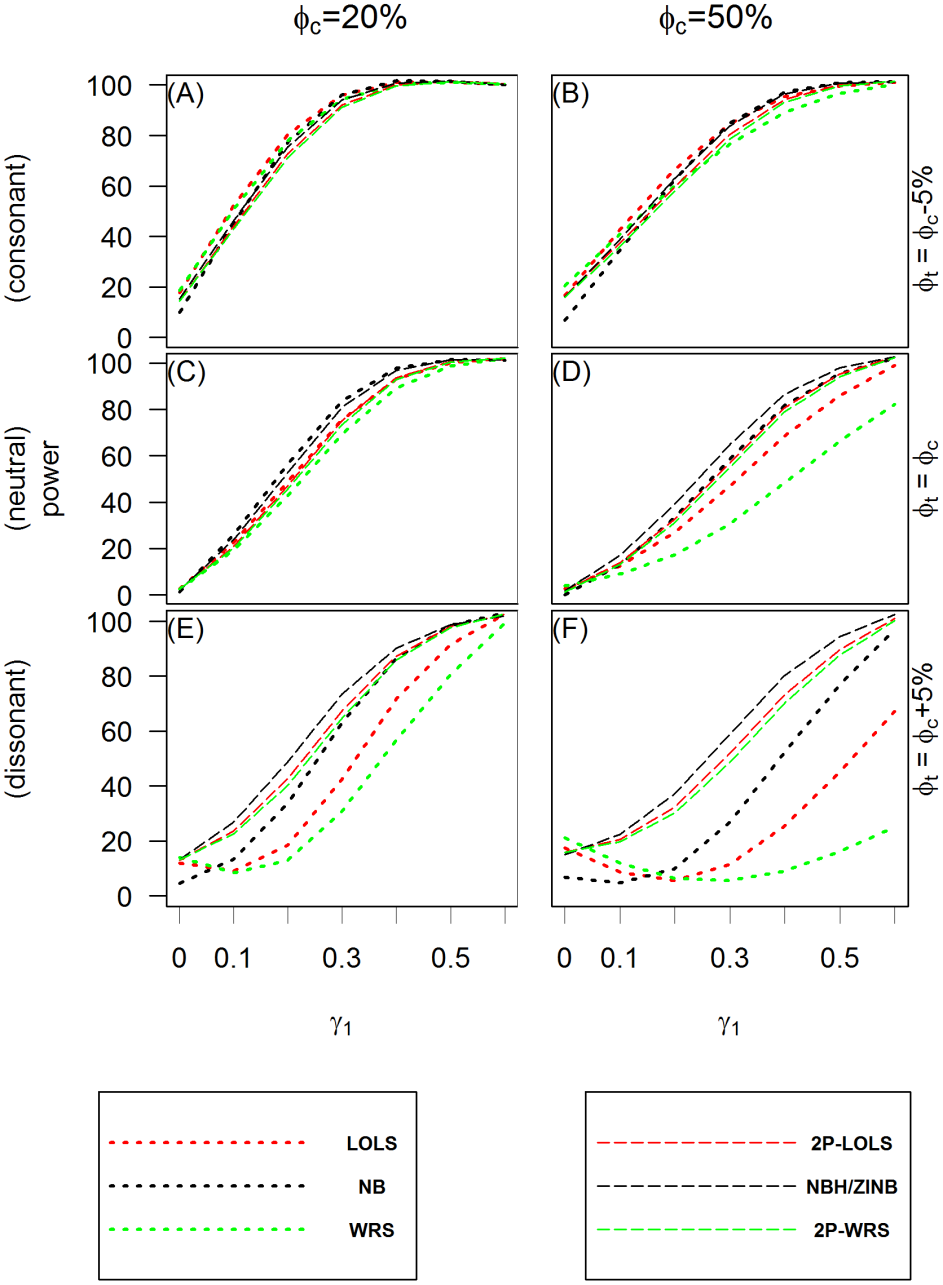
The power of test for ZINB simulated data. The *X* axis is the value of the covariate effect on the count data *γ*_1_ and the *Y* axis is the power of test when the level of significance is 0.05. Three different cases of covariate effect, i.e., the consonant (*ϕ*_*t*_ = *ϕ*_*c*_ − 5%), neutral (*ϕ*_*t*_ = *ϕ*_*c*_) and dissonant (*ϕ*_*t*_ = *ϕ*_*c*_ + 5%) effect, are presented in panels **(A)** and **(B)**; **(C)** and **(D)**; and **(E)** and **(F)**, respectively. Each column reflects different proportion of zero inflation in the unexposed group: 20% in **(A)**, **(C)** and **(E)**; and 50% in **(B)**, **(D)** and **(F)** from the left to the right column, respectively.

Fig 3 and Fig 4 show the power of test when applying different analysis methods to the simulated ZIP and ZINB distributed data, respectively. Methods having the potential of large inflated type I errors (e.g., Poisson model or PH/ZIP model for ZINB distributed data) are not included in these comparisons. These plots show that the hurdle or ZI models perform consistently well in all scenarios examined, while the behaviors of one part models vary across different methods and simulation scenarios. In the consonant effect case, one part models such as LOLS and NB tend to do as well as ZI or hurdle models with WRS performing worse when the proportion of zeros is large. However, in dissonant effect cases, one part models fail to have good power to detect the significance of the overall covariate effect. This is consistent with the observation by Lachenbruch [17] for the continuous non-negative responses with excess zeros. In the neutral effect case, when the proportion of structural zeros is 50% or more, the one-part models also have lower power than the two part models.

### Estimation of the covariate effects

#### Covariate effect *γ*_1_

**Fig 5:**
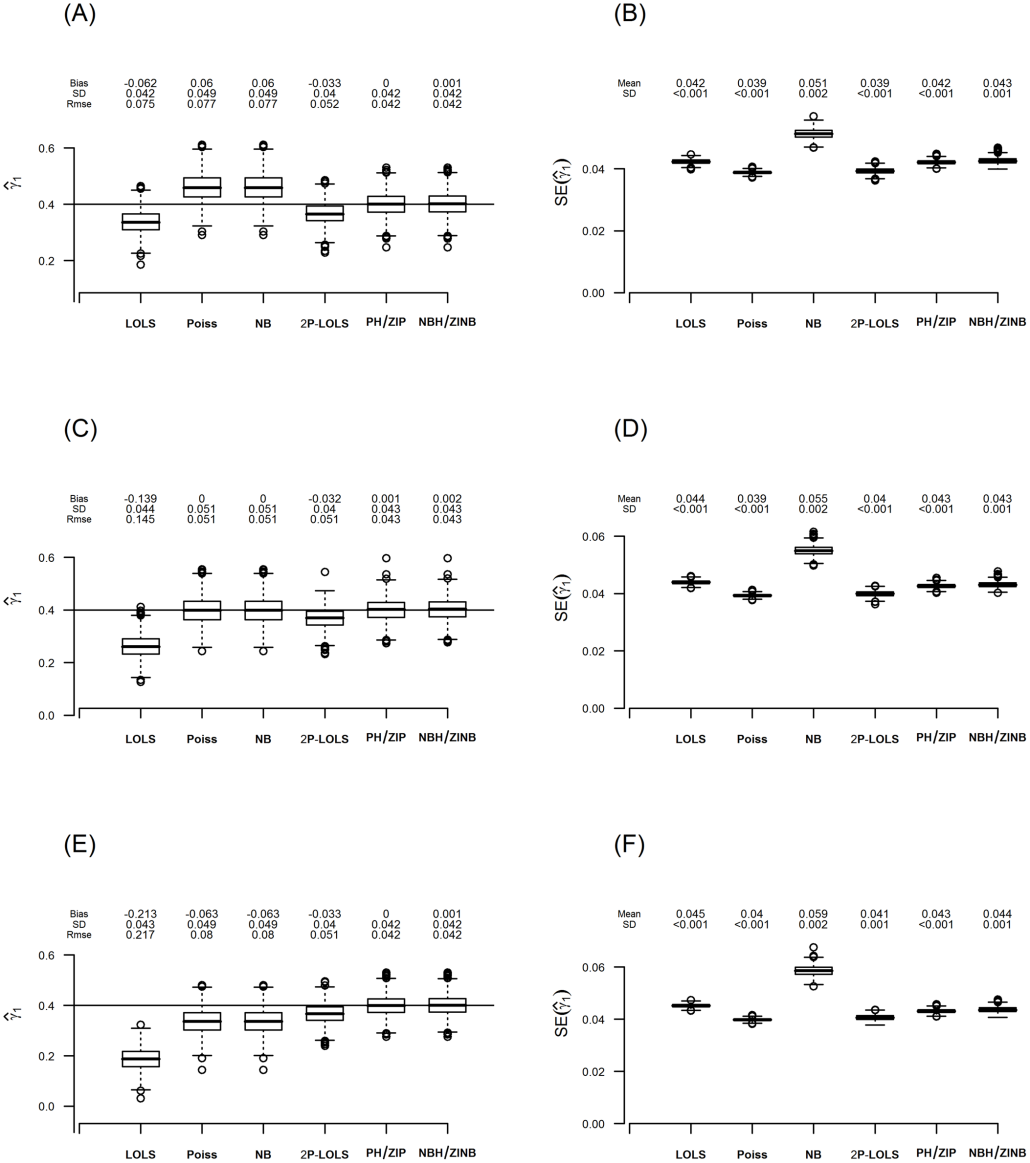
The estimate of *γ*_1_ and its standard error for data simulated under ZIP with ***ϕ***_**c**_ = **20%**. The figure displays box-plots of estimates and their standard errors for *γ*_1_ from 1000 replications in **(A)** and **(B)**; **(C)** and **(D)**; and **(E)** and **(F)** for the consonant (*ϕ*_*t*_ = *ϕ*_*c*_ − 5%), neutral (*ϕ*_*t*_ = *ϕ*_*c*_) and dissonant (*ϕ*_*t*_ = *ϕ*_*c*_ + 5%) effect case, respectively. For each box of the boxplots, the center line represents the median, the bottom line represents the 25th percentiles and the top line represents the 75th percentiles. The whiskers of the boxplots show 1.5 interquartile range (IQR) below the 25th percentiles and 1.5 IQR above the 75th percentiles, and outliers are represented by small circles. The horizontal line in **(A)**, **(C)** and **(E)** represents the true value of *γ*_1_ (= 0.4) and the bias, standard deviation (SD), and root mean square error (RMSE) of the estimations of *γ*_1_ are shown above its box-plot for each method. The mean and standard deviation (SD) of the standard error (SE) estimations are shown above the box-plot for each method in panels **(B)**, **(D)** and **(F)**.

**Fig 6:**
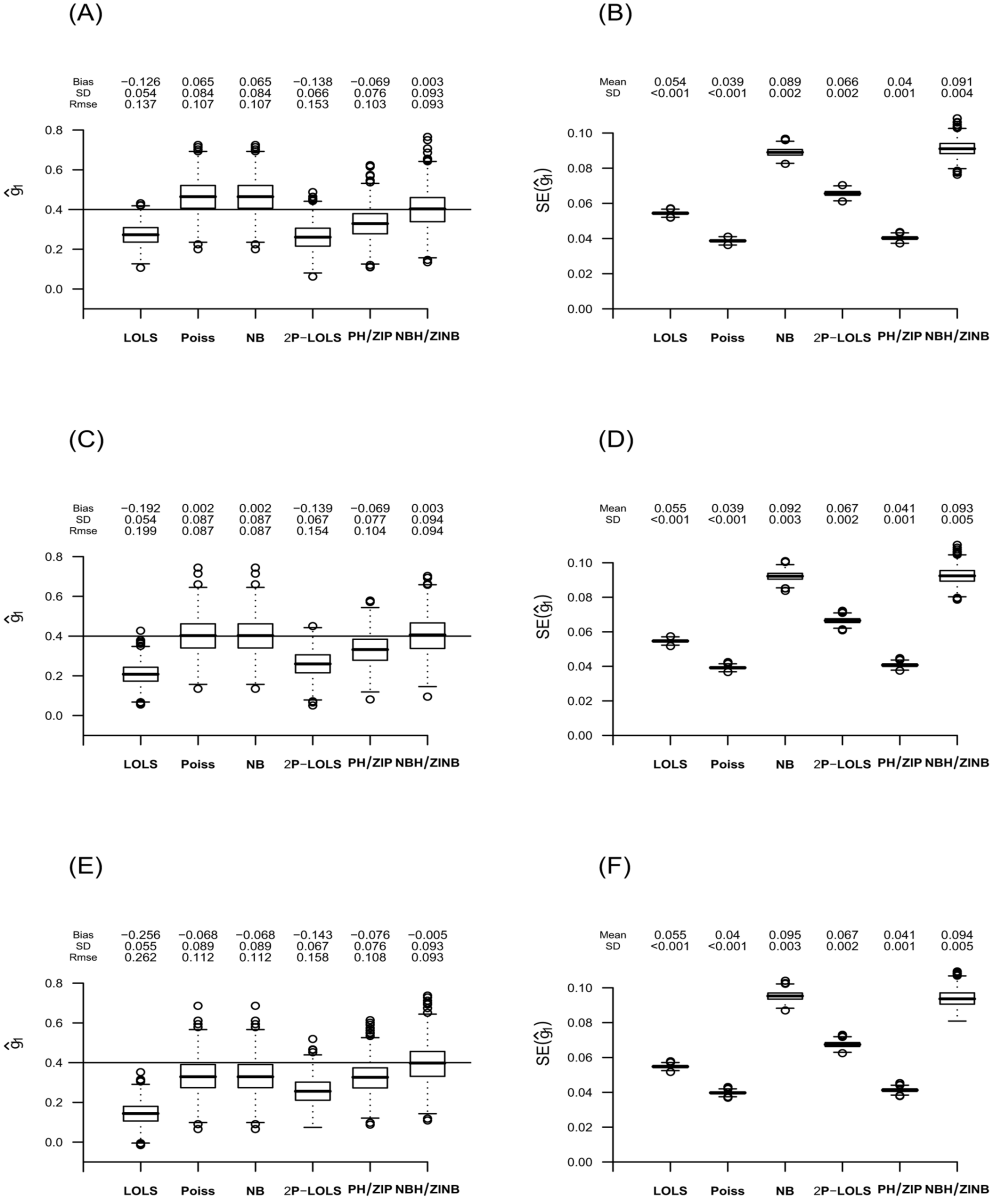
The estimate of *γ*_1_ and its standard error for data simulated under ZINB with ***ϕ***_**c**_ = **20%**. The figure displays box-plots of estimates and their standard errors for *γ*_1_ from 1000 replications in **(A)** and **(B)**; **(C)** and **(D)**; and **(E)** and **(F)** for the consonant (*ϕ*_*t*_ = *ϕ*_*c*_ − 5%), neutral (*ϕ*_*t*_ = *ϕ*_*c*_) and dissonant (*ϕ*_*t*_ = *ϕ*_*c*_ + 5%) effect case, respectively. For each box of the boxplots, the center line represents the median, the bottom line represents the 25th percentiles and the top line represents the 75th percentiles. The whiskers of the boxplots show 1.5 interquartile range (IQR) below the 25th percentiles and 1.5 IQR above the 75th percentiles, and outliers are represented by small circles. The horizontal line in **(A)**, **(C)** and **(E)** represents the true value of *γ*_1_ (= 0.4) and the bias, standard deviation (SD), and root mean square error (RMSE) of the estimations of *γ*_1_ are shown above its box-plot for each method. The mean and standard deviation (SD) of the standard error (SE) estimations are shown above the box-plot for each method in panels **(B)**, **(D)** and **(F)**.

We first examine the covariate effect estimates and their SEs on the log scale of count data levels *γ*_1_. Fig 5 and Fig 6 are the box plots of the estimation results of *γ*_1_ and their standard errors (SEs) for ZIP and ZINB distributed data, respectively, when the true proportion of inflated zeros for unexposed group is 20%and the true value of *γ*_1_ is equal to 0.4. Notice that for every method investigated, the standard deviation (SD) of the estimations for ZINB distributed data are larger than those for ZIP distributed data.

For both ZIP and ZINB distributed data, the pattern of estimation bias of one part models varies across different scenarios. For example, Poisson and NB have unbiased estimation in neutral effect case, but over-estimate in consonant effect and under-estimate in the dissonant effect scenario. LOLS under-estimates in all scenarios, but the absolute value of bias increases as the scenario changes from consonant to neutral, and then to dissonant effect case. The estimation performances of one part models for higher degree of zero inflation show similar patterns (S1 Fig, S2 Fig, and S3 Fig).

On the other hand, the performance of the hurdle and ZI models are consistent across different covariate effect scenarios and different degrees of zero inflation. For ZIP distributed data, both PH/ZIP and ZINB/NBH give unbiased estimation of *γ*_1_. For ZINB distributed data, only NBH/ZINB give unbiased estimation, while PH/ZIP show under-estimation. 2P-LOLS shows improvement than LOLS, but still under-estimates the parameter especially for ZINB distributed data.

We also compare the SE estimation with the sample SD of the estimations. The estimation for SE is significantly deflated for the Poisson method. Deflation in SE can also be seen in PH/ZIP method for ZINB distributed data. On the other hand, NB over-estimates SE, although to a lesser degree. For the ZIP distributed data with 80% zero inflation, NB has some outliers in the estimation of SE, showing unstableness of this method for a high-degree of zero inflation. The SE estimations for other methods are similar to the sample SDs of the estimates. The consequence of the incorrect SE estimation is wrong calculation of p-value and the misleading conclusion about the significance effect test. For example, under-estimated SE can result in enlarged Z value and consequently smaller p-value. On the other hand, over-estimation of SE can yield incorrectly larger p-value.

#### Covariate effect on the probability of (structural) zeros

**Fig 7:**
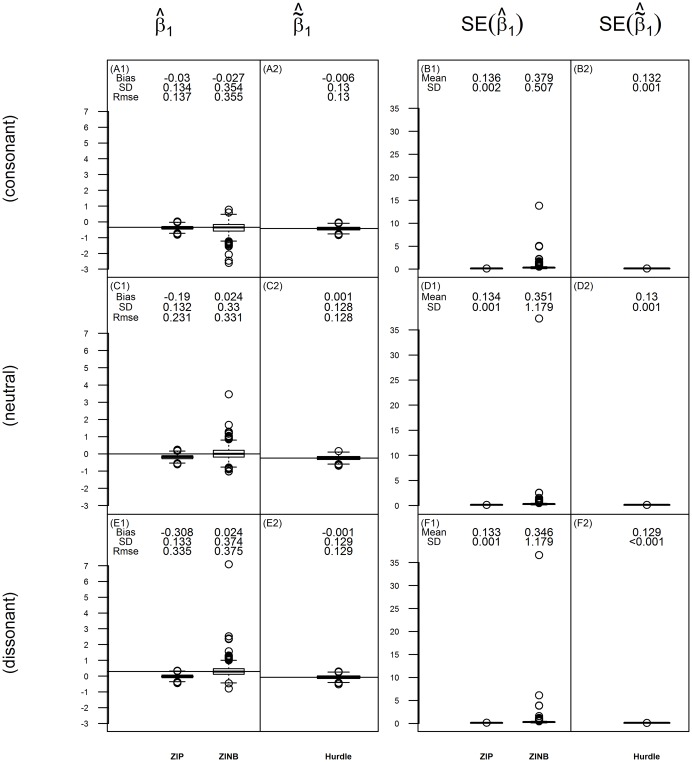
The estimate of *β*_1_ (or ${\tilde{\beta }}_{1}$) and its standard error for data simulated under ZINB when ***ϕ***_**c**_ = **20%** and **γ**_**1**_ = **0.4**. The figure displays box-plots of estimates and their standard errors for the covariate effect on the log-odds of structural zeros for ZIP and ZINB method and on the log-odds of zeros for hurdle models from 1000 replications when *γ*_1_ = 0.4. For each box of the boxplots, the center line represents the median, the bottom line represents the 25th percentiles and the top line represents the 75th percentiles. The whiskers of the boxplots show 1.5 interquartile range (IQR) below the 25th percentiles and 1.5 IQR above the 75th percentiles, and outliers are represented by small circles. Panels **(A1)**, **(C1)** and **(E1)** show the estimates of *β*_1_ for consonant, neutral and dissonant effect case, respectively. The horizontal line in these panels represents the true value of *β*_1_, which is −0.349 in **(A1)**, 0 in **(C1)** and 0.287 in **(E1)**. Panels **(A2)**, **(C2)** and **(E2)** show the estimates of ${\tilde{\beta }}_{1}$ for consonant, neutral and dissonant effect case, respectively. The horizontal line in these panels represents the true value of ${\tilde{\beta }}_{1}$, which is −0.420 in **(A2)**, −0.240 in **(C2)** and −0.070 in **(E2)**. The bias, standard deviation (SD), and root mean square error (RMSE) of the estimates are shown above the box-plot for each method. Panel **(B1)**, **(D1)** and **(F1)** show the SEs of the estimates for *β*_1_, and panel **(B2)**, **(D2)** and **(F2)** show the SEs of the estimates for ${\tilde{\beta }}_{1}$. The mean and standard deviation (SD) of the standard error (SE) estimations are shown above the box-plot for each method.

Fig 7 shows the boxplots of estimations and their SEs for *β*_1_ using ZI models and for ${\tilde{\beta }}_{1}$ using hurdle models when ZINB simulated data has 20% zero inflation in the unexposed group and *γ*_1_ = 0.4. The true values of ${\tilde{\beta }}_{1}$ are derived from the parameter estimations of the ZI model using Equation 1 and 2. Results for other simulation settings are shown in S4 Fig, S5 Fig, S6 Fig, S7 Fig. Because the logistic regression part is the same, the estimations for ${\tilde{\beta }}_{1}$ are identical across different hurdle models. Similarly to the case of *γ*_1_, ZINB has unbiased estimation of *β*_1_ for both ZIP and ZINB distributed data, while ZIP is only unbiased for ZIP distributed data. Notice that when the proportion of zero inflation is low (e.g., *ϕ*_*c*_ = 20%), ZINB may have unstable results with some large SE. The estimations are more stable when the zero inflation proportion increases to 50% or when the sample size is increased (results not given). On the contrary, hurdle models give unbiased and stable estimates for ${\tilde{\beta }}_{1}$ in all simulation scenarios.

### AIC values

Table 2 shows the mean of the AICs from simulations under ZINB distribution when ***ϕ***_**c**_ = **20%**. Results for other settings of *ϕ*_*c*_ and for ZIP distributed data are shown in S1 Table, S2 Table, S3 Table and S4 Table. Not surprisingly, the true underlying model performs the best with the smallest AIC values for each simulation scenario. For ZIP distributed data, the AICs of NBH/ZINB are very close to those of PH/ZIP. However, for ZINB distributed data, PH/ZIP has much larger AICs than the true model. Except in the case of fitting PH/ZIP to ZINB distributed data, hurdle/ZI models in general have smaller AICs than one-part models. Among all the one-part models, NB has the smallest AIC values and for ZINB distributed data with relatively small proportion (e.g., 20%) of excess zeros, it shows better performance than 2P-LOLS.

**Table 2:** The AIC’s of different methods for data simulated under ZINB distribution with *ϕ*_c_ = 20%.

parameters		One part models			Hurdle/ZI models		
*ϕ*_*t*_	*γ*_1_	LOLS	Poisson	NB	2P-LOLS	PH/ZIP	NBH/ZINB
15%	0	4065	5351	3962	3972	4406	**3956**
	0.2	4237	5749	4125	4137	4682	**4118**
	0.6	4609	6733	4472	4491	5384	**4463**
20%	0	4017	5331	3899	3908	4333	**3892**
	0.2	4189	5730	4062	4072	4599	**4054**
	0.6	4552	6730	4395	4408	5258	**4381**
25%	0	3965	5299	3832	3838	4248	**3822**
	0.2	4135	5698	3991	3996	4501	**3979**
	0.6	4490	6722	4313	4320	5134	**4294**

The numbers are the mean of the AIC’s for 1000 replications. *ϕ*_*c*_ is the probability of *y* coming from structural zeros for the unexposed group. *ϕ*_*t*_ is the probability of *y* coming from structural zeros for the exposed group. The smallest AIC values among all fitting models are displayed in bold font.

### Evaluation of model selection procedure

**Fig 8:**
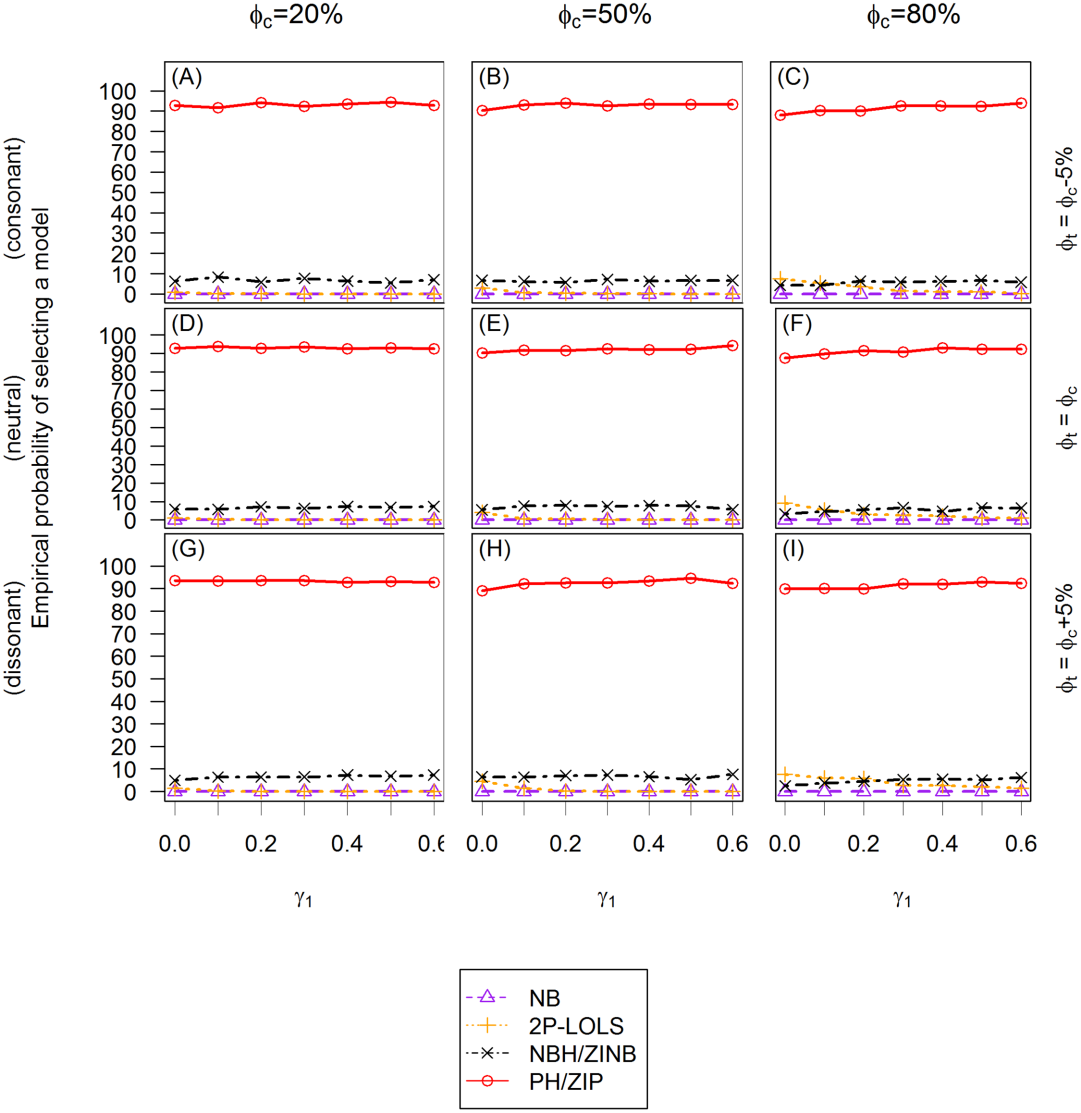
The empirical probability of choosing a model using AIC criterion for ZIP distributed data. The *X* axis is the value of the covariate effect on the count data *γ*_1_ and the *Y* axis is the empirical probability of choosing a model using AIC criterion. Three different cases of covariate effect, i.e., the consonant (*ϕ*_*t*_ = *ϕ*_*c*_ − 5%), neutral (*ϕ*_*t*_ = *ϕ*_*c*_) and dissonant (*ϕ*_*t*_ = *ϕ*_*c*_ + 5%) effect, are presented in **(A)**, **(B)** and **(C)**; **(D)**, **(E)** and **(F)**; and **(G)**, **(H)** and **(I)**, respectively. Each column reflects different proportion of zero inflation in the unexposed group: 20% in **(A)**, **(D)** and **(G)**; 50% in **(B)**, **(E)** and **(H)**; and 80% in **(C)**, **(F)** and **(I)** from the first to the third column.

**Fig 9:**
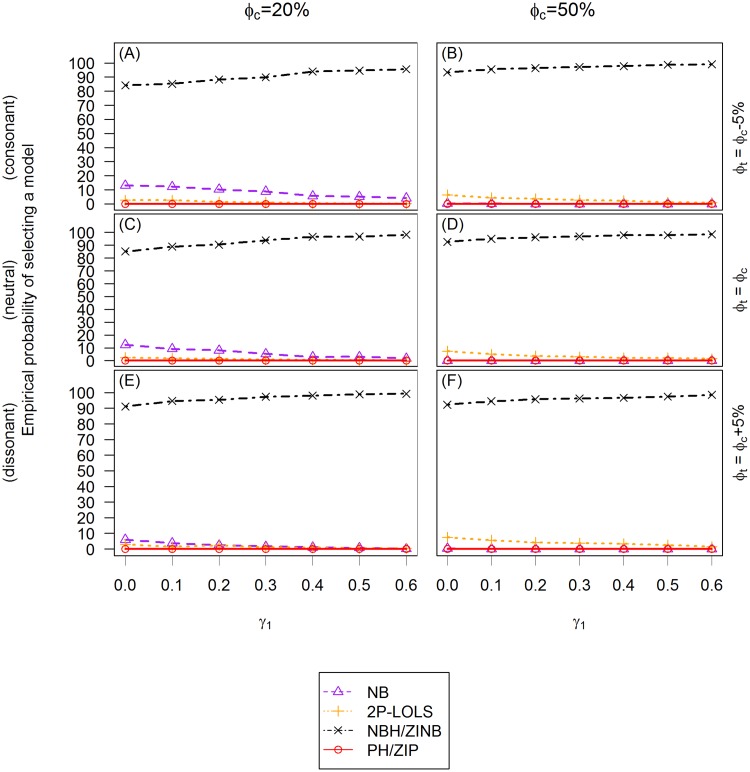
The empirical probability of choosing a model using AIC criterion for ZINB distributed data. The *X* axis is the value of the covariate effect on the count data *γ*_1_ and the *Y* axis is the empirical probability of choosing a model using AIC criterion. Three different cases of covariate effect, i.e., the consonant (*ϕ*_*t*_ = *ϕ*_*c*_ − 5%), neutral (*ϕ*_*t*_ = *ϕ*_*c*_) and dissonant (*ϕ*_*t*_ = *ϕ*_*c*_ + 5%) effect, are presented in **(A)** and **(B)**; **(C)** and **(D)**; and **(E)** and **(F)**, respectively. Each column reflects different proportion of zero inflation in the unexposed group: 20% in **(A)**, **(C)** and **(E)**; and 50% in **(B)**, **(D)** and **(F)** from the left to the right column, respectively.

We examine the ability to select the correct model based on AICs. We also evaluate the performance of Vuong test in the testing of ZINB vs. NB model for the ZINB distributed data. We illustrate the empirical probability of selecting different models using AIC criterion in Fig 8 and Fig 9. Notice that in these simulation studies, because of the binary covariate setting, a ZI model and its corresponding hurdle model have identical AIC values. However, AIC values can be different if continuous covariates are involved [27].

The AIC criterion never selects LOLS nor Poisson models in either ZIP or ZINB distributed data, therefore only NB, 2P-LOLS, PH/ZIP, and NBH/ZINB models are compared. For ZIP distributed data, the plots show that the empirical probabilities of identifying the correct model (i.e., PH/ZIP) are similar across different simulation settings and are around 90%. At about 3–10% of the time, the AIC criterion favors NBH/ZINB model. When *γ*_1_ is small (e.g., < 0.2) and the degree of zero inflation is relatively large, 2P-LOLS is chosen at about 3–15% of the time as the best model. However, if *γ*_1_ is sufficiently large, the chance of choosing the 2P-LOLS becomes rare. The AIC criterion never identifies the ZIP distributed data as NB distributed. For ZINB distributed data, 85- 99% of the time, the AIC model selection procedure will identify the correct distribution (i.e., NBH/ZINB). The probability of the correct identification has smallest value at *γ*_1_ = 0 when *ϕ*_*c*_ = 20%, but increases with the increasing of *γ*_1_ value and the zero inflation degree. When *ϕ*_*c*_ = 20%, the AIC performance has some slight discrepancies among different covariate effect scenarios, with the dissonant effect case having the largest, and the consonant effect case having the smallest correct model identification percentage. The most common mis-specified model for the ZINB distributed data is NB (3–15% of the time) when *ϕ*_*c*_ = 20%, and 2P-LOLS (1–7% of the time) when *ϕ*_*c*_ = 50%. PH/ZIP is never identified as the best model for ZINB distributed data.

Examination of the Vuong test of ZINB vs. NB for the ZINB distributed data when *ϕ*_*c*_ = 20% shows that Vuong test has lower power than AIC criterion in the selection of correct model (results not given in plots or tables). The result shows that about 60% of the time, the Vuong test favors ZINB over NB when *γ*_1_ = 0. Similar to the AIC criterion, the percentage of the correct model identification increases with the increasing of *γ*_1_ value. When *ϕ*_*c*_ = 50%, more than 96% of the time the Vuong test will select the correct model.

### Application to human microbiome study

The specific objective of the Genetic Environmental Microbial (GEM) project is to define the risk factors that lead to the onset of Crohn′s disease through the study of individuals before they develop the disease. Healthy first degree relatives of people with Crohn′s disease, predominantly siblings and offspring, are recruited. Each subject provides a stool sample and bacterial DNA is extracted. The V4 hypervariable region of bacterial 16S rRNA gene are sequenced in paired-end modules (2 × 150 bp) on Illumina MiSeq platform. The resulting paired reads are assembled using paired-end assembler for Illumina sequences PANDAseq v2.7 [35] to generate an amplicon size of 253 base pairs. Assembled reads are demultiplexed and analyzed using Quantitative Insights into Microbial Ecology (QIIME) software v1.8 [36]. For quality filtering, the default parameters of QIIME are maintained. Chimeric sequences are identified and removed using usearch61 [37]. To identify OTUs from the non-chimeric sequences we use a closed reference-based picking approach using UCLUST software against Greengenes database 13_8 of bacterial 16S rRNA sequences. The abundance of a specific bacterial genus can be obtained by aggregating all the counts of assigned sequences to this genus.

In this paper, we choose three organisms to represent the range of overall percentage of zeros, in which Anaerotruncus has a small proportion of zero counts (18%), Dehalobacterium is intermediate (50%) and Campylobacter is high (77%). Histograms for the abundance of these bacteria (S8 Fig) all exhibit right skewed and over-dispersion. There are 204 males and 262 females, and it is of interest to determine whether there is a significant sex difference in the abundance of each of these bacteria. A two sample t-test shows that the mean age of males (19.2 years) is significantly younger than that of females (21.2 years, *p* = 0.006). Therefore, age is included as an additional covariate in the model to adjust for possible confounding. The total number of reads varies among subjects with a mean of 71,490. (SD = 32,839, S9 Fig.).

**Fig 10:**
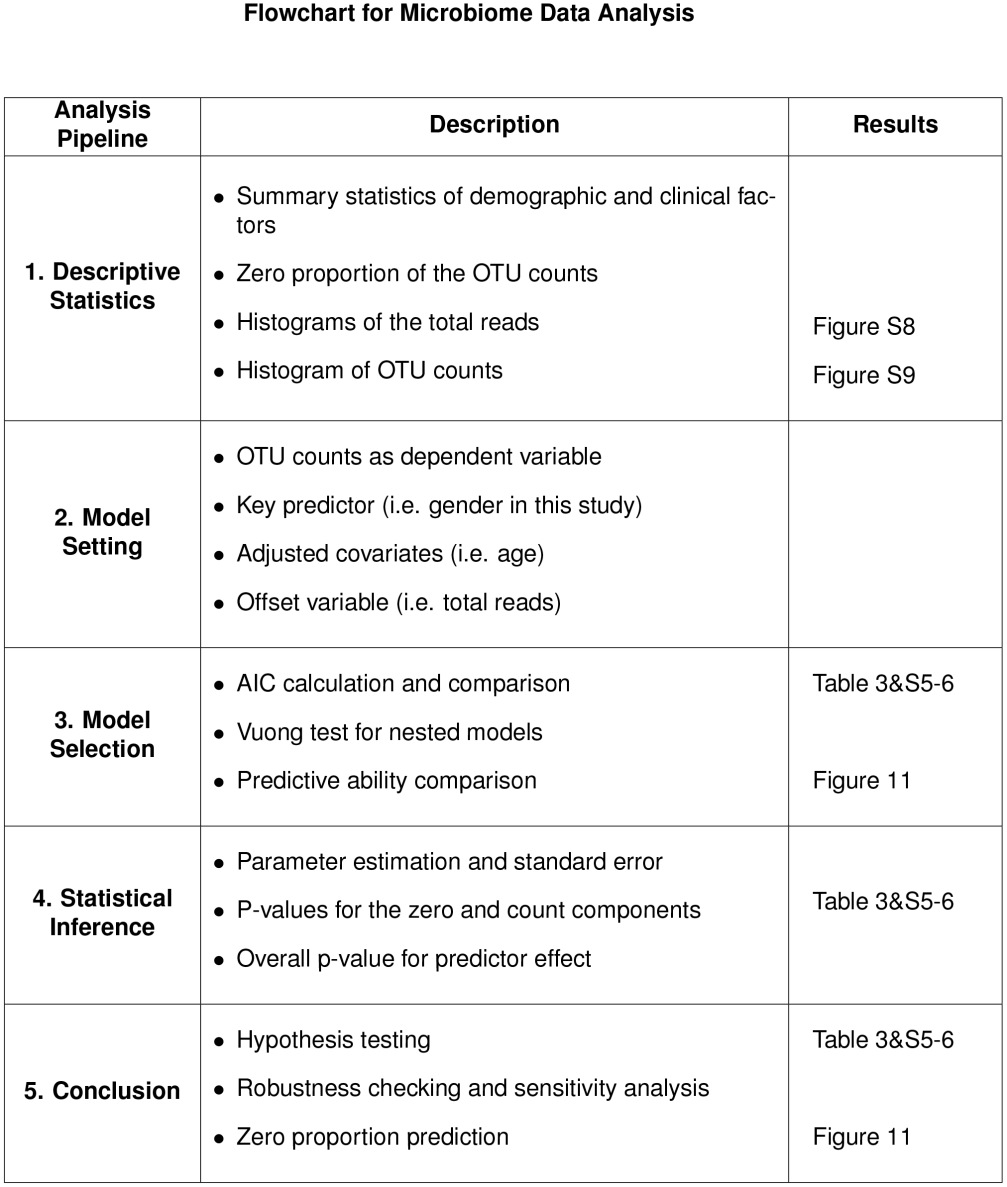
The flowchart for microbiome real data analysis.

We fit the data using the different models discussed, including both gender and age as covariates. For the hurdle/ZI models, they are also covariates for the zero component. We choose female as the reference category for gender. Considering the variation in the total number of sequence counts across samples, we use the log-transformed total number of reads as an offset in a log linear regression model for LOLS, Poisson, NB models, and for the count component of the hurdle/ZI models such as 2P-LOLS, PH, NBH, ZIP and ZINB. We also use it as an offset for the logistic regression part of hurdle models. Results are shown in Table 3 for Campylobacter, and in S5 Table and S6 Table Tables for Anaerotruncus and Dehalobacterium. The flowchart of the data analysis is shown in Fig 10.

**Table 3:** The parameter estimate of the gender effect and goodness of fit for bacteria Campylobacter (proportion of zeros: 77%) using different methods. Female is the reference category for gender.

Model	Logit*		Count distribution		overall	AIC
	*β*_1_ (SE)	*Pr*(> ∣*t*∣)	*γ*_1_ (SE)	*Pr*(> ∣*t*∣)	p-value**	
LOLS	NA	NA	−0.074 (0.074)	0.316	0.316	1388
Poisson	NA	NA	−0.782 (0.091)	< 10^−6^	< 10^−6^	2781
**NB**	NA	NA	**−0.841 (0.306)**	**0.006**	**0.006**	**976^†^**
WRS	NA	NA	NA	NA	0.420	NA
2P-LOLS	0.335(0.236)	0.156	0.002 (0.220)	0.992	0.365	1051
PH	0.320(0.236)	0.174	−0.598 (0.096)	< 10^−6^	< 10^−6^	1792
ZIP	0.226(0.237)	0.342	−0.599 (0.096)	< 10^−6^	< 10^−6^	1793
NBH	0.320(0.236)	0.174	−0.923 (0.470)	0.049	0.059	978^††^
ZINB	0.022(3.567)	0.995	−0.813 (0.410)	0.047	0.047	981^†††^
2P-WRS	NA	NA	NA	NA	0.597	NA

The standard errors (SEs) of estimations are in parentheses. The first, second and third smallest AIC value among different models (except logistic regression) are displayed with superscript ^†^, ^††^, and ^†††^ respectively. The model with its name in bold font is the final selected model.

*: $logit(\phi_{i})=\text{log}(\frac{\phi_{i}}{1-\phi_{i}})=X_{i}^{T}\beta$, where *ϕ* is the probability of zeros/structural zeros as defined in hurdle/ZI models.

**: The overall p-value is the same as the p-value for the one part model. For the hurdle/ZI models, p-value is computed uisng the likelihood ratio test statistics in testing *H*_0_: *β*_1_ = 0, *γ*_1_ = 0 vs. *H*_*A*_: not both are equal to 0.

**Fig 11:**
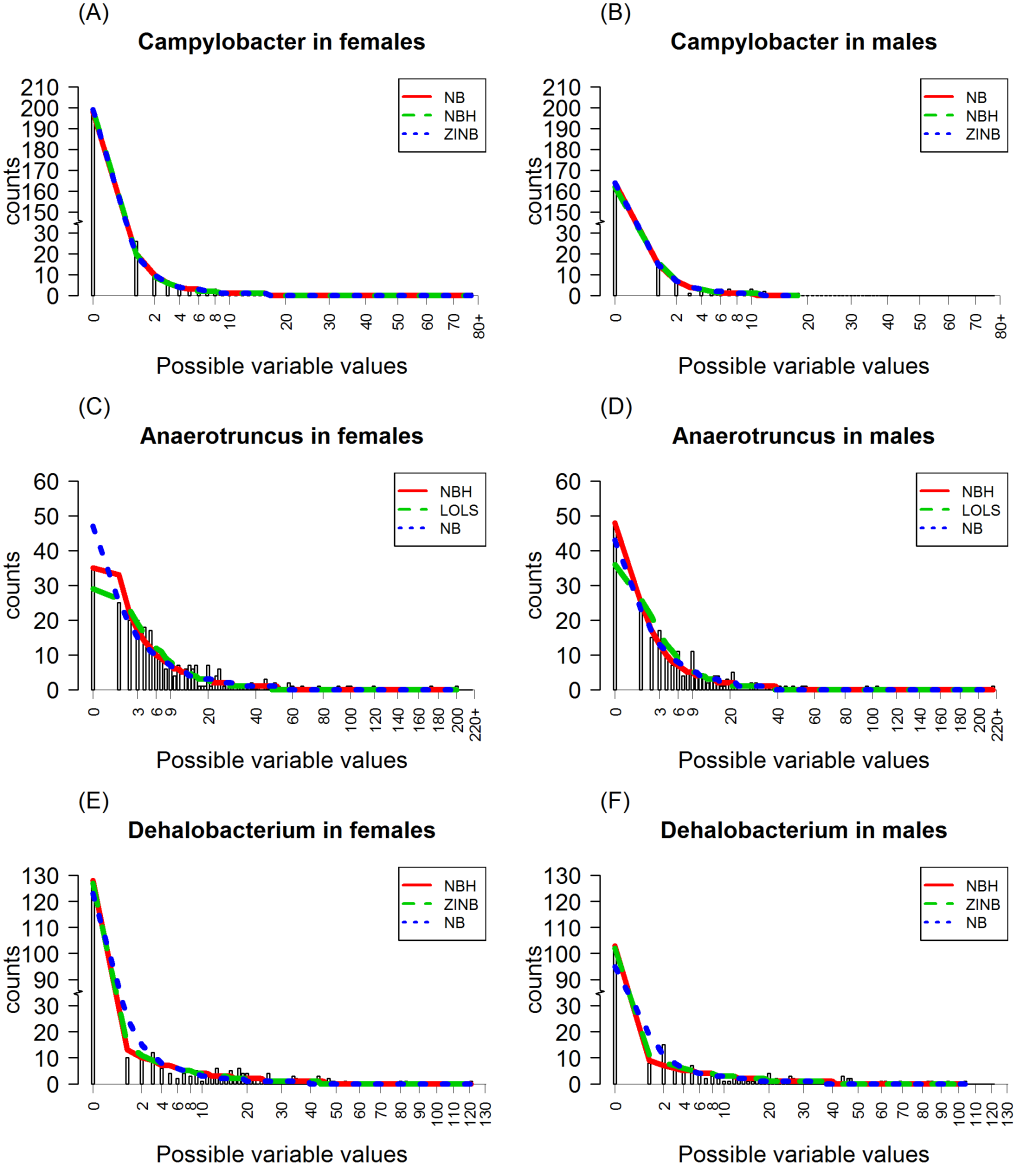
The comparison plots of the observed and expected counts of bacteria for Campylobacter, Anaerotruncus and Dehalobacterium for females and males using the best three models judging by AIC criterion. The *X* axis is the possible values of the OTUs, the bars are the observed counts, the red line connects the expected counts produced by the model with smallest AIC values, the green line connects the expected counts produced by the model with the second smallest AIC values and the blue line connects the expected counts produced by the model with the third smallest AIC values. The first, second and third row of the plots are for bacteria Campylobacter, Anaerotruncus, and Dehalobacterium, respectively.

For Campylobacter with 77% zeros (Table 3), NB, NBH and ZINB has the first, second and third smallest AICs, respectively, and the AIC values are very close. In addition, all of these models consistently detect a significant gender effect, while other models do not. Furthermore, their predictions (Fig 11) are similar and can describe the observed sequence counts very well. They also perform about the same in the estimations of *γ*_1_. However, the ZINB provides a relatively large SE for ${\hat{\beta }}_{1}$, indicating the lack of stability of the parameter estimate in the ZINB parameterization. Vuong test shows no particular preference for any of these three models. We thus choose NB as the fitting model and conclude that gender is significantly associated with the OTU count levels of Campylobacter, with males having significantly lower mean counts than females.

For bacteria Anaerotruncus, with 18% of zeros, all models consistently suggest significant association of its abundance with gender, but not age (Poisson family models excluded). Among all the fitted models, NBH, LOLS and NB has the first, second and third smallest AIC values, respectively. Vuong test favors NBH over NB and ZINB (*p* < = 0.05), but no preference between NB and ZINB. Fig 11 shows that these three models perform similarly on the prediction of the counts of 2 or more. However, NB appears to overestimate the probability of zero counts for females but underestimates it for males. LOLS underestimates zero counts for both females and males. For NBH, the predicted probability of zero counts matches the observed probability. Notice that ZINB has a large SE (= 24.705) for ${\hat{\beta }}_{1}$ suggesting non-convergence of the ZINB model. Therefore we choose NBH as the most appropriate model and conclude that there is a significant association between gender and the abundance of bacteria Anaerotruncus. This association is through the effect of gender on the probability of zero OTU counts, with males having higher chance of having zero counts.

For Dehalobacterium, with 50% of zeros, NBH, ZINB and NB has the first, second and third smallest AICs, respectively. Vuong test has the same order of model favor (*p* < 0.01). We thus choose NBH as the most appropriate model for the OTU counts of this bacteria. Note that the predictions from ZINB and NBH model are indistinguishable and they describe the data better than NB model (Fig 11). An inspection of the fitting results using formula $p(y\in \{0\})=\frac{\text{exp}(\beta_{0}+\beta_{1}Sex+\beta_{2}Age)}{1+\text{exp}(\beta_{0}+\beta_{1}Sex+\beta_{2}Age)}$ shows that the proportion of structural zeros identified by a ZINB model is about 40%. If we are interested in modeling these structural zeros, then the ZINB model should be used instead. Notice that all models suggest insignificant gender effect on the sequence counts, and we thus conclude that there is no significant association between gender and the abundance of Dehalobacterium.

## Discussion

In microbiome research, count data with excess zeros is commonly encountered. To assess the importance of accounting for zero inflation and the consequence of mis-specifying the statistical models, we designed a comprehensive simulation study and compared the performance of different competing methods under a variety of scenarios such as different degrees of zero inflation, different directions of covariate effect on the structural zero and count components, and variation of the count component from equi- to over-dispersion. We focused on the assessment of type I error, power to detect overall covariate effect, measures of model fit, and bias and effectiveness of parameter estimations.

Results confirm that if the data is zero inflated, standard one part models in general will fail to provide a good model fit, which may result in biased and inefficient parameter estimations and the possibility of type I error or loss of power. Particularly, Poisson regression has substantially inflated type I errors and thus is not suitable for count data with excess zeros. On the contrary, NB has less type I errors than expected and may be prone to reduced power. Although LOLS has well-controlled type I error rates and competitive power in the consonant case, it is not robust to other situations of covariate effects. Furthermore, one part models tend to under-estimate the frequencyies of zeros and have biased estimation of the covariate effect size, which may result in incorrect sample size estimation needed for replication studies.

The ZIP and ZINB models were specifically developed for count data with structural zeros. Among the two ZI models, ZINB is more robust since it can handle both over- and equi-dispersion in the count component, while ZIP is only suitable for the later scenario. The ZI regressions allow us to investigate not only the possible association of environmental or genetic factors with the count levels, but also their associations with the probability of structural zeros.

A drawback of the ZI models is that they may have non-convergence or local maxima problems due to their computation complexity as a result of simultaneous estimation of both the structural zero and count components [19, 38]. Consequently, the parameter estimations of the structural zero component may be unstable. In contrast, hurdle models (PH or NBH) provide more stable parameter estimations for both the zero and count components and they are robust if there is no zero inflation and can handle zero deflation problems [25]. Furthermore, the fitting indices (such as AICs) and the estimation of the effect for the magnitude of the count data from the corresponding hurdle and ZI models are similar. Therefore, due to the computational consideration, if the interest is in prediction or if the data-generating mechanism of the zeros is unknown, we suggest to choose PH or NBH (depending whether over-dispersion is present in the count component) over the ZI models. However, if the study goal is in statistical inference, the model choice should also be adjusted by clinical reasoning [23]. If structural zeros are believed to exist and the interest is in modeling them, the ZI models should be chosen. In this case, if non-convergence is encounter, then a larger sample size is probably required.

Other hurdle models such as 2P-LOLS and 2P-WRS show relatively comparable power to the true ZIP or ZINB model and have well-controlled type I errors in testing the overall covariate effect. Furthermore, they are robust for the case of over-dispersion in the count component and thus can be considered if we are just interested in the testing of association. However, due to its mis-specification of the counts as the continuous normally distributed data, 2P-LOLS will in general result in biased parameter estimation of the covariate effects and is thus not recommended for statistical inference on the bacterial counts. 2P-WRS cannot be used for statistical inference either.

As this simulation study has shown, the inappropriate application of a statistical model could have undesirable consequences. Therefore, it is important for researchers to perform model selection to choose the most appropriate model. Our simulation confirms that the AIC criterion has good power in identifying the correct distribution of the data and Vuong test has less power. However, caution should be given for the possibility of model mis-identification using these selection strategies. For example, for the ZINB distributed data with relatively low degree of zero inflation, it is possible that NB is mis-identified as the best model. Therefore, a graphical examination of the comparison of the observed with the predicted values is recommended.

In this paper, we focus on the discussion of the simulation results for binary covariate case. Additional simulations are also conducted (results not given) for a continuous covariate and we have similar observations as in the case of a binary covariate. Particularly, although not exactly identical, the AIC value produced by NBH and ZINB model are very close and are the smallest among all fitted models. We also evaluate the overall type I error and power of test for two other commonly used models: OLS and logistic regression (Results are provided in S7 Table, S10 Fig and S11 Fig). Both OLS and logistic regression have well-controlled type I error rates. It is interesting to see that OLS performs well in terms of power in the consonant effect scenario. However, similar to other one-part models, it is not robust to other scenarios (i.e., dissonant or neutral effects). The supplementary material also provides the evaluation results of relative bias of prediction for zeros for the competing models (S8 Table, S9 Table, S10 Table, S11 Table and S12 Table). All the one part models underestimate the probability of the zeros while the hurdle and ZI count models (i.e., PH, ZIP, NBH, ZINB) show unbiased estimations. 2P-LOLS has small bias, and the bias decreases when the proportion of inflated zeros becomes higher.

Our simulation study just focuses on the independent data assumption, however, in clinical studies and the microbiome field, observations maybe serial or related say due to families, thus in future work we will extend our evaluation to the related data with excess zeros.

## Supporting Information

S1 Table The AIC’s of different methods for data simulated under ZIP distribution with ***ϕ***_c_ = 20%. The numbers are the mean of the AIC’s for 1000 replications. *ϕ*_*c*_ is the probability of *y* coming from structural zeros for the non-exposed group. *ϕ*_*t*_ is the probability of *y* coming from structural zeros for the exposed group. The smallest AIC values among all fitting models are displayed in bold font.
(PDF)

S2 Table The AIC’s of different methods for data simulated under ZIP distribution with ***ϕ***_c_ = 50%. The numbers are the mean of the AIC’s for 1000 replications. *ϕ*_*c*_ is the probability of *y* coming from structural zeros for the non-exposed group. *ϕ*_*t*_ is the probability of *y* coming from structural zeros for the exposed group. The smallest AIC values among all fitting models are displayed in bold font.
(PDF)

S3 Table The AIC’s of different methods for data simulated under ZIP distribution with ***ϕ***_c_ = 80%. The numbers are the mean of the AIC’s for 1000 replications. *ϕ*_*c*_ is the probability of *y* coming from structural zeros for the non-exposed group. *ϕ*_*t*_ is the probability of *y* coming from structural zeros for the exposed group. The smallest AIC values among all fitting models are displayed in bold font.
(PDF)

S4 Table The AIC’s of different methods for data simulated under ZINB distribution with ***ϕ*>**_c_ = 50%. The numbers are the mean of the AIC’s for 1000 replications. *ϕ*_*c*_ is the probability of *y* coming from structural zeros for the non-exposed group. *ϕ*_*t*_ is the probability of *y* coming from structural zeros for the exposed group. The smallest AIC values among all fitting models are displayed in bold font.
(PDF)

S5 TableThe parameter estimate of the gender effect and goodness of fit for bacteria Anaerotruncus (proportion of zeros: 18%) using different methods.Female is the reference category for gender. The standard errors (SEs) of estimations are in parentheses. The first, second and third smallest AIC value among different models (except logistic regression) are displayed with superscript ^†^, ^††^, and ^†††^ respectively. The model with its name in bold font is the final selected model. *: $logit(\phi_{i})=\text{log}(\frac{\phi_{i}}{1-\phi_{i}})=X_{i}^{T}\beta$, where *ϕ* is the probability of zeros/structural zeros as defined in hurdle/ZI models. **: The overall p-value is the same as the p-value for the one part model. For the hurdle/ZI models, p-value is computed uisng the likelihood ratio test statistics in testing *H*_0_: *β*_1_ = 0, *γ*_1_ = 0 vs. *H*_*A*_: not both are equal to 0.(PDF)

S6 TableThe parameter estimate of the gender effect and goodness of fit for bacteria Dehalobacterium (proportion of zeros: 50%) using different methods.Female is the reference category for gender. The standard errors (SEs) of estimations are in parentheses. The first, second and third smallest AIC value among different models (except logistic regression) are displayed with superscript ^†^, ^††^, and ^†††^ respectively. The model with its name in bold font is the final selected model. *: $logit(\phi_{i})=\text{log}(\frac{\phi_{i}}{1-\phi_{i}})=X_{i}^{T}\beta$, where *ϕ* is the probability of zeros/structural zeros as defined in hurdle/ZI models. **: The overall p-value is the same as the p-value for the one part model. For the hurdle/ZI models, p-value is computed uisng the likelihood ratio test statistics in testing *H*_0_: *β*_1_ = 0, *γ*_1_ = 0 vs. *H*_*A*_: not both are equal to 0.(PDF)

S7 Table The type I error rate estimations for different competing models (including OLS and Logistic regression). Estimates are based on 10,000 replicated samples. *ϕ*_*c*_ is the probability of *y* coming from structural zeros for the non-exposed group. *α* is the significant level of test. A bold value represents inflated type I error.
(PDF)

S8 Table The relative bias for P(y = 0) for data simulated under ZIP distribution with ***ϕ***_c_ = 20%. The numbers are the mean of the relative bias of p(y = 0) for 1000 replications. *ϕ*_*c*_ is the probability of *y* coming from structural zeros for the non-exposed group. *ϕ*_*t*_ is the probability of *y* coming from structural zeros for the exposed group.
(PDF)

S9 Table The relative bias for P(y = 0) for data simulated under ZIP distribution with ***ϕ***_c_ = 50%. The numbers are the mean of the relative bias of p(y = 0) for 1000 replications. *ϕ*_*c*_ is the probability of *y* coming from structural zeros for the non-exposed group. *ϕ*_*t*_ is the probability of *y* coming from structural zeros for the exposed group.
(PDF)

S10 Table The relative bias for P(y = 0) for data simulated under ZIP distribution with ***ϕ***_c_ = 80%. The numbers are the mean of the relative bias of p(y = 0) for 1000 replications. *ϕ*_*c*_ is the probability of *y* coming from structural zeros for the non-exposed group. *ϕ*_*t*_ is the probability of *y* coming from structural zeros for the exposed group.
(PDF)

S11 Table The relative bias for P(y = 0) for data simulated under ZINB distribution with ***ϕ***_c_ = 20%. The numbers are the mean of the relative bias of p(y = 0) for 1000 replications. *ϕ*_*c*_ is the probability of *y* coming from structural zeros for the non-exposed group. *ϕ*_*t*_ is the probability of *y* coming from structural zeros for the exposed group.
(PDF)

S12 Table The relative bias for P(y = 0) for data simulated under ZINB distribution with ***ϕ***_c_ = 50%. The numbers are the mean of the relative bias of p(y = 0) for 1000 replications. *ϕ*_*c*_ is the probability of *y* coming from structural zeros for the non-exposed group. *ϕ*_*t*_ is the probability of *y* coming from structural zeros for the exposed group.
(PDF)

S1 Fig The estimate of *γ*_1_ and its standard error for data simulated under ZIP distribution with ***ϕ***_c_ = 50%. The box-plot of *γ*_1_ estimates (in the left column panel) and their corresponding SE estimates (in the right column panel) for 1000 replications of simulated ZIP data using LOLS, Poisson, NB, 2P-LOLS and ZINB methods. The horizontal line in the left column plots is the true value of *γ*_1_, which is 0.4. The consonant, neutral and dissonant scenarios are displayed in the first, second and third rows, respectively. The bias, root mean square error (rmse) and standard deviation (sd) of the estimations of *γ*_1_ are shown above its box-plot for each method in the left column. The mean and standard deviation (sd) of the standard error (SE) estimations above the box-plot for each method in the right column.
(PDF)

S2 Fig The estimate of *γ*_1_ and its standard error for data simulated under ZIP distribution with ***ϕ***_c_ = 80%. The box-plot of *γ*_1_ estimates (in the left column panel) and their corresponding SE estimates (in the right column panel) for 1000 replications of simulated ZIP data using LOLS, Poisson, NB, 2P-LOLS and ZINB methods. The horizontal line in the left column plots is the true value of *γ*_1_, which is 0.4. The consonant, neutral and dissonant scenarios are displayed in the first, second and third rows, respectively. The bias, root mean square error (rmse) and standard deviation (sd) of the estimations of *γ*_1_ are shown above its box-plot for each method in the left column. The mean and standard deviation (sd) of the standard error (SE) estimations above the box-plot for each method in the right column.
(PDF)

S3 Fig The estimate of *γ*_1_ and its standard error for data simulated under ZINB distribution with ***ϕ***_c_ = 50%. The box-plot of *γ*_1_ estimates (in the left column panel) and their corresponding SE estimates (in the right column panel) for 1000 replications of simulated ZINB data using LOLS, Poisson, NB, 2P-LOLS and ZINB methods. The horizontal line in the left column plots is the true value of *γ*_1_, which is 0.4. The consonant, neutral and dissonant scenarios are displayed in the first, second and third rows, respectively. The bias, root mean square error (rmse) and standard deviation (sd) of the estimations of *γ*_1_ are shown above its box-plot for each method in the left column. The mean and standard deviation (sd) of the standard error (SE) estimations above the box-plot for each method in the right column.
(PDF)

S4 FigThe estimate of *β*_1_ (or ${\tilde{\text{β}}}_{1}$) and its standard error for data simulated under ZIP distribution with ***ϕ***_c_ = 20%.The figure displays box-plots of estimates and their standard errors for the covariate effect on the log-odds of structural zeroes for ZIP and ZINB method and on the log-odds of zeroes for hurdle models from 1000 replications. Panels **(A1)**, **(C1)**, and **(E1)** show the estimates of *β*_1_ for consonant, neutral and dissonant effect case, respectively. The horizontal line in these panels represents the true value of *β*_1_, which is −0.349 in **(A1)**, 0 in **(C1)** and 0.287 in **(E1)**. Panels **(A2)**, **(C2)**, and **(E2)** show the estimates of ${\tilde{\beta }}_{1}$ for consonant, neutral and dissonant effect case, respectively. The horizontal line in these panels represents the true value of ${\tilde{\beta }}_{1}$, which is −0.540 in **(A2)**, −0.218 in **(C2)** and 0.053 in **(E2)**. The bias, root mean square error (RMSE) and standard deviation (SD) of the estimates are shown above the box-plot for each method. Panel **(B1)**, **(D1)**, and **(F1)** show the SEs of the estimates for *β*_1_, and panel **(B2)**, **(D2)**, and **(F2)** show the SEs of the estimates for ${\tilde{\beta }}_{1}$. The mean and standard deviation (SD) of the standard error (SE) estimations are shown above the box-plot for each method.(TIFF)

S5 FigThe estimate of *β*_1_ (or ${\tilde{\text{β}}}_{1}$) and its standard error for data simulated under ZIP distribution with ***ϕ***_c_ = 50%.The figure displays box-plots of estimates and their standard errors for the covariate effect on the log-odds of structural zeroes for ZIP and ZINB method and on the log-odds of zeroes for hurdle models from 1000 replications. Panels **(A1)**, **(C1)**, and **(E1)** show the estimates of *β*_1_ for consonant, neutral and dissonant effect case, respectively. The horizontal line in these panels represents the true value of *β*_1_, which is −0.201 in **(A1)**, 0 in **(C1)** and 0.201 in **(E1)**. Panels **(A2)**, **(C2)**, and **(E2)** show the estimates of ${\tilde{\beta }}_{1}$ for consonant, neutral and dissonant effect case, respectively. The horizontal line in these panels represents the true value of ${\tilde{\beta }}_{1}$, which is −0.295 in **(A2)**, −0.098 in **(C2)** and 0.100 in **(E2)**. The bias, standard deviation (SD), and root mean square error (RMSE) of the estimates are shown above the box-plot for each method. Panel **(B1)**, **(D1)**, and **(F1)** show the SEs of the estimates for *β*_1_, and panel **(B2)**, **(D2)**, and **(F2)** show the SEs of the estimates for ${\tilde{\beta }}_{1}$. The mean and standard deviation (SD) of the standard error (SE) estimations are shown above the box-plot for each method.(TIFF)

S6 FigThe estimate of *β*_1_ (or ${\tilde{\text{β}}}_{1}$) and its standard error for data simulated under ZIP distribution with ***ϕ***_c_ = 80%.The figure displays box-plots of estimates and their standard errors for the covariate effect on the log-odds of structural zeroes for ZIP and ZINB method and on the log-odds of zeroes for hurdle models from 1000 replications. Panels **(A1)**, **(C1)**, and **(E1)** show the estimates of *β*_1_ for consonant, neutral and dissonant effect case, respectively. The horizontal line in these panels represents the true value of *β*_1_, which is −0.287 in **(A1)**, 0 in **(C1)** and 0.349 in **(E1)**. Panels **(A2)**, **(C2)**, and **(E2)** show the estimates of ${\tilde{\beta }}_{1}$ for consonant, neutral and dissonant effect case, respectively. The horizontal line in these panels represents the true value of ${\tilde{\beta }}_{1}$, which is −0.349 in **(A2)**, −0.063 in **(C2)** and 0.285 in **(E2)**. The bias, standard deviation (SD), and root mean square error (RMSE) of the estimates are shown above the box-plot for each method. Panel **(B1)**, **(D1)**, and **(F1)** show the SEs of the estimates for *β*_1_, and panel **(B2)**, **(D2)**, and **(F2)** show the SEs of the estimates for ${\tilde{\beta }}_{1}$. The mean and standard deviation (SD) of the standard error (SE) estimations are shown above the box-plot for each method.(TIFF)

S7 FigThe estimate of *β*_1_ (or ${\tilde{\text{β}}}_{1}$) and its standard error for data simulated under ZINB distribution with ***ϕ***_c_ = 50%.The figure displays box-plots of estimates and their standard errors for the covariate effect on the log-odds of structural zeroes for ZIP and ZINB method and on the log-odds of zeroes for hurdle models from 1000 replications. Panels **(A1)**, **(C1)**, and **(E1)** show the estimates of *β*_1_ for consonant, neutral and dissonant effect case, respectively. The horizontal line in these panels represents the true value of *β*_1_, which is −0.201 in **(A1)**, 0 in **(C1)** and 0.201 in **(E1)**. Panels **(A2)**, **(C2)**, and **(E2)** show the estimates of ${\tilde{\beta }}_{1}$ for consonant, neutral and dissonant effect case, respectively. The horizontal line in these panels represents the true value of ${\tilde{\beta }}_{1}$, which is −0.315 in **(A2)**, −0.151 in **(C2)** and 0.020 in **(E2)**. The bias, standard deviation (SD), and root mean square error (RMSE) of the estimates are shown above the box-plot for each method. Panel **(B1)**, **(D1)**, and **(F1)** show the SEs of the estimates for *β*_1_, and panel **(B2)**, **(D2)**, and **(F2)** show the SEs of the estimates for ${\tilde{\beta }}_{1}$. The mean and standard deviation (SD) of the standard error (SE) estimations are shown above the box-plot for each method.(TIFF)

S8 Fig The histogram of of the abundance for bacteria Anaerotruncus, Dehalobacterium and Campylobacter. The X-axis is the possible counts of the bacterium in the square root scale. The Y-axis is the frequency of the counts with some line breaks.
(PDF)

S9 Fig The histogram of total number of sequence counts for the bacteria classified at genus level. The red dashed line represent the mean of the total counts (71,490) and the blue dotted line represent the median of the total counts (65,438). The range is from 13,647 to 196,591. The standard deviation of the total counts is 32,839.
(PDF)

S10 Fig The power of test for ZIP simulated data for competing models (including OLS and logistic regression). The *X* axis is the value of the covariate effect on the count data *γ*_1_ and the *Y* axis is the power of test when the level of significance is 0.05. Three different cases of covariate effect, i.e., the consonant (*ϕ*_*t*_ = *ϕ*_*c*_ − 5%), neutral (*ϕ*_*t*_ = *ϕ*_*c*_) and dissonant (*ϕ*_*t*_ = *ϕ*_*c*_ + 5%) effect, are presented in {**(A), (B), (C)**}, {**(D), (E), (F)**}, and {**(G), (H), (I)**}, respectively. Each column reflects different proportion of zero inflation in the non-exposed group: 20% in {**(A), (D), (G)**}, 50% in {**(B), (E), (H)**} and 80% in {**(C), (F), (I)**} from the first to the third column.
(TIFF)

S11 Fig The power of test for ZINB simulated data for competing models (including OLS and logistic regression). The *X* axis is the value of the covariate effect on the count data *γ*_1_ and the *Y* axis is the power of test when the level of significance is 0.05. Three different cases of covariate effect, i.e., the consonant (*ϕ*_*t*_ = *ϕ*_*c*_ − 5%), neutral (*ϕ*_*t*_ = *ϕ*_*c*_) and dissonant (*ϕ*_*t*_ = *ϕ*_*c*_ + 5%) effect, are presented in {**(A), (B)**}, {**(C), (D)**}, and {**(E), (F)**}, respectively. Each column reflects different proportion of zero inflation in the non-exposed group: 20% in {**(A), (C), (E)**} and 50% in {**(B), (D), (F)**} from the left to the right column, respectively.
(TIFF)
