# Supplementary material for: Assessment and Selection of Competing Models for Zero-Inflated Microbiome Data
Source: PLoS One. 2015 Jul 6;10(7):e0129606. doi: 10.1371/journal.pone.0129606 (PMC4493133; doi:10.1371/journal.pone.0129606)
Supplement: S1 Table — The numbers are the mean of the AIC’s for 1000 replications. ϕ c is the probability of y coming from structural zeros for the non-exposed group. ϕ t is the probability of y coming from structural zeros for the exposed group. The smallest AIC values among all fitting models are displayed in bold font. (PDF) [file pone.0129606.s001.pdf]

The AIC's of different methods for data simulated under ZIP distribution with $\phi_c = 20\%$.

parameters		One part models			Hurdle/ZI models		
ϕ_t	γ_1	LOLS	Poisson	NB	2P-LOLS	PH/ZIP	NBH/ZINB
15%	0	3963	3958	3859	3815	**3773**	3774
	0.2	4134	4134	4016	3955	**3905**	3907
	0.6	4522	4545	4358	4214	**4145**	4146
20%	0	3950	3974	3846	3791	**3749**	3750
	0.2	4129	4167	4009	3927	**3878**	3880
	0.6	4532	4635	4365	4178	**4113**	4114
25%	0	3930	3983	3825	3756	**3716**	3718
	0.2	4110	4194	3990	3886	**3840**	3841
	0.6	4516	4704	4350	4125	**4062**	4064
