# Supplementary material for: Assessment and Selection of Competing Models for Zero-Inflated Microbiome Data
Source: PLoS One. 2015 Jul 6;10(7):e0129606. doi: 10.1371/journal.pone.0129606 (PMC4493133; doi:10.1371/journal.pone.0129606)
Supplement: S2 Table — The numbers are the mean of the AIC’s for 1000 replications. ϕ c is the probability of y coming from structural zeros for the non-exposed group. ϕ t is the probability of y coming from structural zeros for the exposed group. The smallest AIC values among all fitting models are displayed in bold font. (PDF) [file pone.0129606.s002.pdf]

The AIC's of different methods for data simulated under ZIP distribution with $\phi_c = 50\%$.

parameters		One part models			Hurdle/ZI models		
ϕ_t	γ_1	LOLS	Poisson	NB	2P-LOLS	PH/ZIP	NBH/ZINB
45%	0	3472	3804	3267	3129	**3101**	3103
	0.2	3645	4065	3421	3244	**3212**	3213
	0.6	4013	4718	3723	3432	**3388**	3389
50%	0	3411	3762	3184	3047	**3020**	3022
	0.2	3573	4025	3325	3150	**3120**	3121
	0.6	3932	4699	3616	3329	**3287**	3288
55%	0	3337	3700	3090	2951	**2926**	2928
	0.2	3496	3968	3226	3050	**3022**	3023
	0.6	3835	4643	3494	3210	**3170**	3172
