# Supplementary material for: Assessment and Selection of Competing Models for Zero-Inflated Microbiome Data
Source: PLoS One. 2015 Jul 6;10(7):e0129606. doi: 10.1371/journal.pone.0129606 (PMC4493133; doi:10.1371/journal.pone.0129606)
Supplement: S3 Table — The numbers are the mean of the AIC’s for 1000 replications. ϕ c is the probability of y coming from structural zeros for the non-exposed group. ϕ t is the probability of y coming from structural zeros for the exposed group. The smallest AIC values among all fitting models are displayed in bold font. (PDF) [file pone.0129606.s003.pdf]

The AIC's of different methods for data simulated under ZIP distribution with $\phi_c = 80\%$.

parameters		One part models			Hurdle/ZI models		
ϕ_t	γ_1	LOLS	Poisson	NB	2P-LOLS	PH/ZIP	NBH/ZINB
75%	0	2401	2663	1870	1778	**1766**	1767
	0.2	2526	2892	1947	1834	**1820**	1821
	0.6	2808	3513	2105	1932	**1911**	1913
80%	0	2274	2497	1713	1631	**1620**	1621
	0.2	2390	2718	1783	1682	**1669**	1671
	0.6	2642	3283	1921	1764	**1746**	1748
85%	0	2134	2302	1548	1473	**1463**	1464
	0.2	2235	2497	1608	1517	**1506**	1507
	0.6	2439	2979	1707	1567	**1553**	1554
