# Supplementary material for: Assessment and Selection of Competing Models for Zero-Inflated Microbiome Data
Source: PLoS One. 2015 Jul 6;10(7):e0129606. doi: 10.1371/journal.pone.0129606 (PMC4493133; doi:10.1371/journal.pone.0129606)
Supplement: S4 Table — The numbers are the mean of the AIC’s for 1000 replications. ϕ c is the probability of y coming from structural zeros for the non-exposed group. ϕ t is the probability of y coming from structural zeros for the exposed group. The smallest AIC values among all fitting models are displayed in bold font. (PDF) [file pone.0129606.s004.pdf]

The AIC's of different methods for data simulated under ZINB distribution with $\phi_c = 50\%$.

parameters		One part models			Hurdle/ZI models		
ϕ_t	γ_1	LOLS	Poisson	NB	2P-LOLS	PH/ZIP	NBH/ZINB
45%	0	3336	4694	3016	3006	3282	**2996**
	0.2	3490	5092	3144	3133	3480	**3121**
	0.6	3817	6120	3403	3389	3962	**3371**
50%	0	3267	4617	2926	2916	3182	**2906**
	0.2	3406	4988	3036	3025	3350	**3013**
	0.6	3723	5999	3281	3265	3794	**3248**
55%	0	3177	4501	2813	2801	3049	**2792**
	0.2	3324	4883	2933	2919	3224	**2908**
	0.6	3620	5872	3155	3135	3632	**3120**
