# Supplementary material for: Assessment and Selection of Competing Models for Zero-Inflated Microbiome Data
Source: PLoS One. 2015 Jul 6;10(7):e0129606. doi: 10.1371/journal.pone.0129606 (PMC4493133; doi:10.1371/journal.pone.0129606)
Supplement: S5 Table — Female is the reference category for gender. The standard errors (SEs) of estimations are in parentheses. The first, second and third smallest AIC value among different models (except logistic regression) are displayed with superscript †, ††, and ††† respectively. The model with its name in bold font is the final selected model. *: logit(ϕi)=log(ϕi1−ϕi)=XiTβ, where ϕ is the probability of zeros/structural zeros as defined in hurdle/ZI models. **: The overall p-value is the same as the p-value for the one part model. For the hurdle/ZI models, p-value is computed uisng the likelihood ratio test statistics in testing H 0: β 1 = 0, γ 1 = 0 vs. H A: not both are equal to 0. (PDF) [file pone.0129606.s005.pdf]

The parameter estimate of the gender effect and goodness of fit for bacteria Anaerotruncus (proportion of zeros: 18%) using different methods.

Model	Logit*		Count distribution		P-value for	AIC
	β_1 (SE)	$Pr(>\|t\|)$	γ_1 (SE)	$Pr(>\|t\|)$	overall effect**	
LOLS	NA	NA	-0.368 (0.107)	6.48×10^{-4}	6.48×10^{-4}	3082††
Poisson	NA	NA	-0.371 (0.029)	$< 10^{-6}$	$< 10^{-6}$	9950
NB	NA	NA	-0.330 (0.129)	0.010	0.010	3083†††
WRS	NA	NA	NA	NA	0.002	NA
2P-LOLS	0.730(0.251)	0.004	-0.238 (0.120)	0.047	0.002	3140
PH	0.715(0.252)	0.005	-0.260 (0.029)	$< 10^{-6}$	1.78×10^{-4}	8748
ZIP	0.710 (0.252)	0.005	-0.260 (0.029)	$< 10^{-6}$	1.76×10^{-4}	8770
**NBH**	**0.715(0.252)**	**0.005**	**-0.237 (0.157)**	**0.130**	**0.006**	**3075 †**
ZINB	7.260 (24.705)	0.769	-0.298 (0.137)	0.029	0.034	3089
2P-WRS	NA	NA	NA	NA	0.005	NA
