# Supplementary material for: Assessment and Selection of Competing Models for Zero-Inflated Microbiome Data
Source: PLoS One. 2015 Jul 6;10(7):e0129606. doi: 10.1371/journal.pone.0129606 (PMC4493133; doi:10.1371/journal.pone.0129606)
Supplement: S7 Table — Estimates are based on 10,000 replicated samples. ϕ c is the probability of y coming from structural zeros for the non-exposed group. α is the significant level of test. A bold value represents inflated type I error. (PDF) [file pone.0129606.s007.pdf]

The type I error rate estimations for different competing models (including OLS and Logistic regression).

	ZIP distributed data						ZINB distributed data			
ϕ_c	20%		50%		80%		20%		50%	
α	.05	.10	.05	.10	.05	.10	.05	.10	.05	.10
OLS	.049	.104	.05	.103	.051	.104	.049	.101	.051	.102
LOLS	.052	.101	.051	.105	.054	.104	.052	.101	.050	.099
Poisson	**.117**	**.193**	**.205**	**.287**	**.273**	**.361**	**.345**	**.422**	**.394**	**.471**
NB	.045	.090	.027	.060	.028	.059	.040	.085	.031	.069
WRS	.050	.102	.052	.105	.054	.106	.053	.103	.050	.099
logistic	.047	.089	.059	.092	.046	.099	.054	.011	.049	.098
2P-TN	.051	.104	.053	.106	.058	.113	.039	.083	.034	.075
2P-LOLS	.050	.103	.053	.110	.060	.113	.054	.104	.051	.100
PH/ZIP	.053	.104	.053	.104	.054	.104	.**219**	.**306**	.**224**	.**309**
NBH/ZINB	.051	.103	.049	.098	.051	.100	.051	.098	.057	.112
2P-WRS	.047	.099	.047	.101	.044	0.094	.053	.103	.049	.098
