# Supplementary material for: Assessment and Selection of Competing Models for Zero-Inflated Microbiome Data
Source: PLoS One. 2015 Jul 6;10(7):e0129606. doi: 10.1371/journal.pone.0129606 (PMC4493133; doi:10.1371/journal.pone.0129606)
Supplement: S8 Table — The numbers are the mean of the relative bias of p(y = 0) for 1000 replications. ϕ c is the probability of y coming from structural zeros for the non-exposed group. ϕ t is the probability of y coming from structural zeros for the exposed group. (PDF) [file pone.0129606.s008.pdf]

The relative bias for P(y=0) for data simulated under ZIP distribution with $\phi_c = 20\%$.

One-part models							
parameters		Non-exposed			Exposed		
ϕ_t	γ_1	LOLS	Poisson	NB	LOLS	Poisson	NB
15%	0	-0.240	-0.549	-0.279	-0.188	-0.517	-0.187
	0.2	-0.221	-0.548	-0.277	-0.313	-0.67	-0.312
	0.6	-0.173	-0.55	-0.263	-0.585	-0.904	-0.583
20%	0	-0.222	-0.549	-0.225	-0.221	-0.549	-0.225
	0.2	-0.197	-0.549	-0.216	-0.328	-0.692	-0.333
	0.6	-0.135	-0.55	-0.179	-0.558	-0.907	-0.553
25%	0	-0.211	-0.552	-0.175	-0.243	-0.564	-0.247
	0.2	-0.173	-0.548	-0.147	-0.333	-0.699	-0.335
	0.6	-0.105	-0.549	-0.093	-0.53	-0.903	-0.519

Hurdle/Zero inflated models							
parameters		Non-exposed			Exposed		
ϕ_t	γ_1	2P-LOLS	PH/ZIP	NBH/ZINB	2P-LOLS	PH/ZIP	NBH/ZINB
15%	0	0.008	0.002	0.002	0.008	0.000	0.000
	0.2	0.006	0.000	0.000	0.000	-0.004	-0.004
	0.6	0.007	0.003	0.003	0.001	0.001	0.001
20%	0	0.004	-0.002	-0.002	0.006	0.000	0.000
	0.2	0.003	-0.003	-0.003	0.001	-0.001	-0.001
	0.6	0.003	-0.001	-0.001	0.000	0.000	0.000
25%	0	-0.001	-0.007	-0.007	0.004	-0.001	-0.001
	0.2	0.008	0.002	0.002	0.002	0.000	0.000
	0.6	0.003	-0.001	-0.001	0.001	0.001	0.001
