# Supplementary material for: Assessment and Selection of Competing Models for Zero-Inflated Microbiome Data
Source: PLoS One. 2015 Jul 6;10(7):e0129606. doi: 10.1371/journal.pone.0129606 (PMC4493133; doi:10.1371/journal.pone.0129606)
Supplement: S9 Table — The numbers are the mean of the relative bias of p(y = 0) for 1000 replications. ϕ c is the probability of y coming from structural zeros for the non-exposed group. ϕ t is the probability of y coming from structural zeros for the exposed group. (PDF) [file pone.0129606.s009.pdf]

The relative bias for P(y=0) for data simulated under ZIP distribution with $\phi_c = 50\%$.

One-part models							
parameters		Non-exposede			Exposed		
ϕ_t	γ_1	LOLS	Poisson	NB	LOLS	Poisson	NB
45%	0	-0.268	-0.515	-0.097	-0.265	-0.536	-0.052
	0.2	-0.262	-0.518	-0.089	-0.322	-0.656	-0.093
	0.6	-0.241	-0.515	-0.054	-0.441	-0.855	-0.177
50%	0	-0.269	-0.516	-0.067	-0.271	-0.518	-0.068
	0.2	-0.261	-0.516	-0.052	-0.319	-0.631	-0.100
	0.6	-0.243	-0.518	-0.017	-0.426	-0.832	-0.173
55%	0	-0.272	-0.517	-0.037	-0.271	-0.49	-0.079
	0.2	-0.263	-0.517	-0.021	-0.316	-0.602	-0.109
	0.6	-0.243	-0.516	0.020	-0.411	-0.803	-0.169

Hurdle/Zero inflated models							
parameters		Non-exposed			Exposed		
ϕ_t	γ_1	2P-LOLS	PH/ZIP	NBH/ZINB	2P-LOLS	PH/ZIP	NBH/ZINB
45%	0	0.003	0.001	0.001	0.004	0.002	0.002
	0.2	0.000	-0.001	-0.001	0.000	0.000	0.000
	0.6	0.003	0.001	0.001	-0.002	-0.002	-0.002
50%	0	0.003	0.001	0.001	0.001	-0.001	-0.001
	0.2	0.003	0.001	0.001	0.001	0.000	0.000
	0.6	0.000	-0.001	-0.001	-0.001	-0.001	-0.001
55%	0	0.002	0.000	0.000	0.002	0.001	0.001
	0.2	0.000	-0.002	-0.002	0.002	0.001	0.001
	0.6	0.001	0.000	0.000	0.000	0.000	0.000
