# Supplementary material for: Assessment and Selection of Competing Models for Zero-Inflated Microbiome Data
Source: PLoS One. 2015 Jul 6;10(7):e0129606. doi: 10.1371/journal.pone.0129606 (PMC4493133; doi:10.1371/journal.pone.0129606)
Supplement: S10 Table — The numbers are the mean of the relative bias of p(y = 0) for 1000 replications. ϕ c is the probability of y coming from structural zeros for the non-exposed group. ϕ t is the probability of y coming from structural zeros for the exposed group. (PDF) [file pone.0129606.s010.pdf]

The relative bias for P(y=0) for data simulated under ZIP distribution with $\phi_c = 80\%$.

One-part models							
parameters		Non-exposed			Exposed		
ϕ_t	γ_1	LOLS	Poisson	NB	LOLS	Poisson	NB
75%	0	-0.241	-0.285	-0.025	-0.25	-0.339	0.005
	0.2	-0.247	-0.283	-0.019	-0.283	-0.424	-0.002
	0.6	-0.263	-0.284	-0.006	-0.351	-0.612	-0.021
80%	0	-0.234	-0.284	-0.007	-0.234	-0.284	-0.007
	0.2	-0.242	-0.286	-0.002	-0.263	-0.360	-0.014
	0.6	-0.258	-0.287	0.011	-0.324	-0.534	-0.030
85%	0	-0.225	-0.283	0.010	-0.217	-0.227	-0.020
	0.2	-0.234	-0.287	0.015	-0.241	-0.289	-0.027
	0.6	-0.246	-0.282	0.031	-0.291	-0.437	-0.039

Hurdle/Zero inflated models							
parameters		Non-exposed			Exposed		
ϕ_t	γ_1	2P-LOLS	PH/ZIP	NBH/ZINB	2P-LOLS	PH/ZIP	NBH/ZINB
75%	0	0.001	0.000	0.000	0.000	-0.001	-0.001
	0.2	0.002	0.001	0.001	0.000	0.000	0.000
	0.6	0.001	0.000	0.000	0.000	0.000	0.000
80%	0	0.001	0.001	0.001	0.001	0.000	0.000
	0.2	0.000	0.000	0.000	0.001	0.001	0.001
	0.6	0.000	-0.001	-0.001	0.000	0.000	0.000
85%	0	0.001	0.001	0.001	0.000	-0.001	-0.001
	0.2	0.000	-0.001	-0.001	0.000	0.000	0.000
	0.6	0.002	0.001	0.001	0.001	0.001	0.001
