# Supplementary material for: Assessment and Selection of Competing Models for Zero-Inflated Microbiome Data
Source: PLoS One. 2015 Jul 6;10(7):e0129606. doi: 10.1371/journal.pone.0129606 (PMC4493133; doi:10.1371/journal.pone.0129606)
Supplement: S11 Table — The numbers are the mean of the relative bias of p(y = 0) for 1000 replications. ϕ c is the probability of y coming from structural zeros for the non-exposed group. ϕ t is the probability of y coming from structural zeros for the exposed group. (PDF) [file pone.0129606.s011.pdf]

The relative bias for P(y=0) for data simulated under ZINB distribution with $\phi_c = 20\%$.

One-part models							
parameters		Non-exposed			Exposed		
ϕ_t	γ_1	LOLS	Poisson	NB	LOLS	Poisson	NB
15%	0	-0.25	-0.721	-0.055	-0.238	-0.736	0.002
	0.2	-0.237	-0.724	-0.055	-0.273	-0.827	-0.008
	0.6	-0.16	-0.726	-0.052	-0.359	-0.948	-0.042
20%	0	-0.250	-0.723	-0.027	-0.252	-0.725	-0.028
	0.2	-0.238	-0.726	-0.025	-0.285	-0.815	-0.041
	0.6	-0.207	-0.723	-0.013	-0.365	-0.941	-0.077
25%	0	-0.251	-0.723	0.002	-0.262	-0.709	-0.054
	0.2	-0.238	-0.726	0.007	-0.293	-0.8	-0.068
	0.6	-0.206	-0.723	0.025	-0.371	-0.933	-0.107

Hurdle/Zero inflated models							
parameters		Non-exposed			Exposed		
ϕ_t	γ_1	2P-LOLS	PH/ZIP	NBH/ZINB	2P-LOLS	PH/ZIP	NBH/ZINB
15%	0	0.022	0.001	0.001	0.023	-0.002	-0.002
	0.2	0.025	0.001	0.001	0.021	-0.001	-0.001
	0.6	0.029	-0.002	-0.002	0.015	-0.001	-0.001
20%	0	0.023	0.002	0.002	0.021	0.000	0.000
	0.2	0.024	0.000	0.000	0.020	0.002	0.002
	0.6	0.033	0.002	0.002	0.016	0.003	0.003
25%	0	0.022	0.001	0.001	0.020	0.001	0.001
	0.2	0.023	-0.001	-0.001	0.019	0.003	0.003
	0.6	0.032	0.001	0.001	0.014	0.003	0.003
