# Supplementary material for: Assessment and Selection of Competing Models for Zero-Inflated Microbiome Data
Source: PLoS One. 2015 Jul 6;10(7):e0129606. doi: 10.1371/journal.pone.0129606 (PMC4493133; doi:10.1371/journal.pone.0129606)
Supplement: S12 Table — The numbers are the mean of the relative bias of p(y = 0) for 1000 replications. ϕ c is the probability of y coming from structural zeros for the non-exposed group. ϕ t is the probability of y coming from structural zeros for the exposed group. (PDF) [file pone.0129606.s012.pdf]

The relative bias for P(y=0) for data simulated under ZINB distribution with $\phi_c = 50\%$.

One-part models							
parameters		Non-exposed			Exposed		
ϕ_t	γ_1	LOLS	Poisson	NB	LOLS	Poisson	NB
45%	0	-0.291	-0.591	-0.033	-0.293	-0.623	0.002
	0.2	-0.286	-0.593	-0.026	-0.322	-0.721	-0.001
	0.6	-0.281	-0.591	-0.020	-0.381	-0.876	-0.024
50%	0	-0.293	-0.592	-0.014	-0.292	-0.592	-0.014
	0.2	-0.288	-0.59	-0.009	-0.321	-0.689	-0.024
	0.6	-0.283	-0.593	0.001	-0.377	-0.853	-0.043
55%	0	-0.293	-0.59	0.006	-0.293	-0.56	-0.03
	0.2	-0.291	-0.594	0.011	-0.317	-0.655	-0.038
	0.6	-0.284	-0.593	0.024	-0.370	-0.824	-0.056

Hurdle/Zero inflated models							
parameters		Non-exposed			Exposed		
ϕ_t	γ_1	2P-LOLS	PH/ZIP	NBH/ZINB	2P-LOLS	PH/ZIP	NBH/ZINB
45%	0	0.009	0.000	0.000	0.008	-0.002	-0.002
	0.2	0.010	-0.001	-0.001	0.018	0.011	0.011
	0.6	0.015	0.002	0.002	0.007	0.001	0.001
50%	0	0.008	0.000	0.000	0.010	0.001	0.001
	0.2	0.011	0.001	0.001	0.005	-0.002	-0.002
	0.6	0.012	-0.001	-0.001	0.005	0.000	0.000
55%	0	0.010	0.001	0.001	0.007	0.000	0.000
	0.2	0.010	0.000	0.000	0.007	0.001	0.001
	0.6	0.013	0.001	0.001	0.005	0.001	0.001
