# Supplementary figures and images for: Assessment and Selection of Competing Models for Zero-Inflated Microbiome Data
Source: PLoS One. 2015 Jul 6;10(7):e0129606. doi: 10.1371/journal.pone.0129606 (PMC4493133; doi:10.1371/journal.pone.0129606)

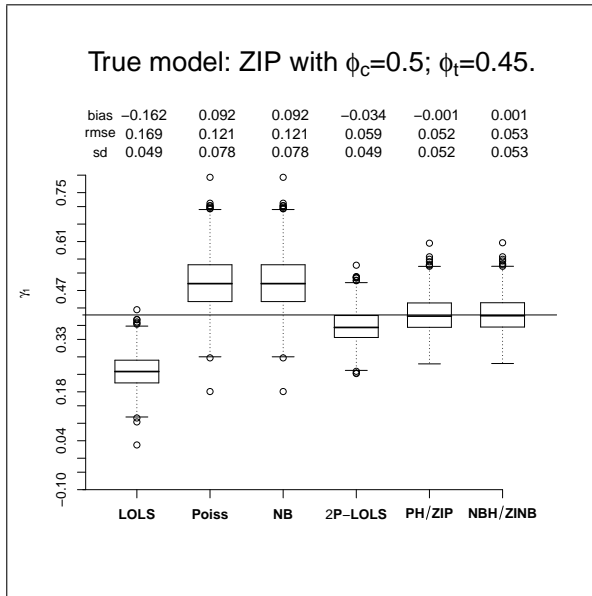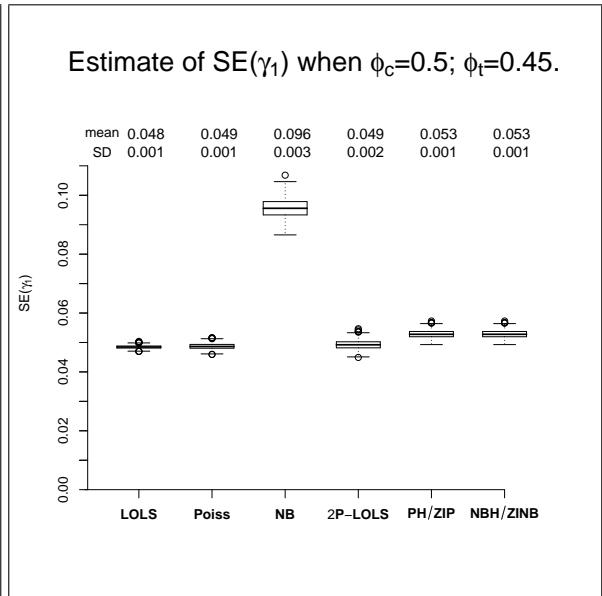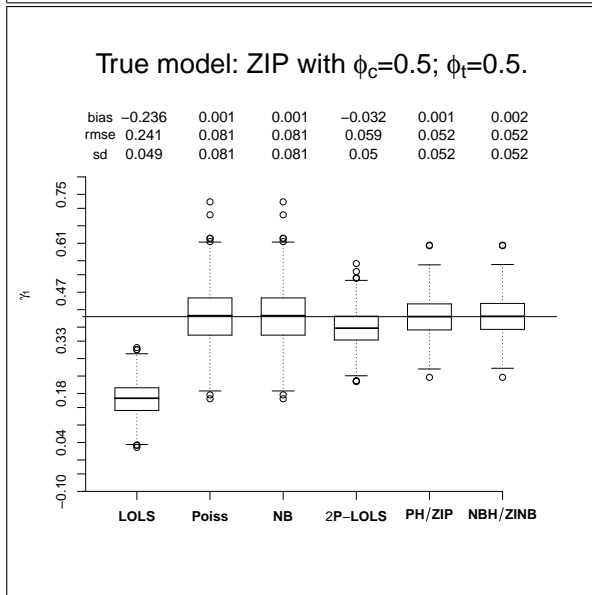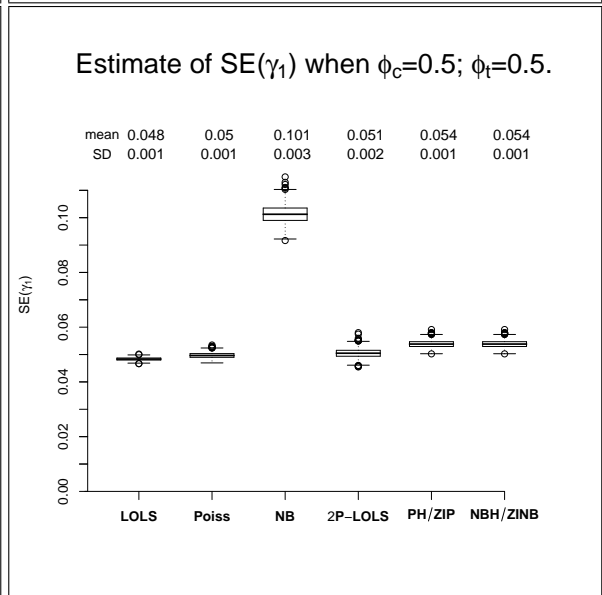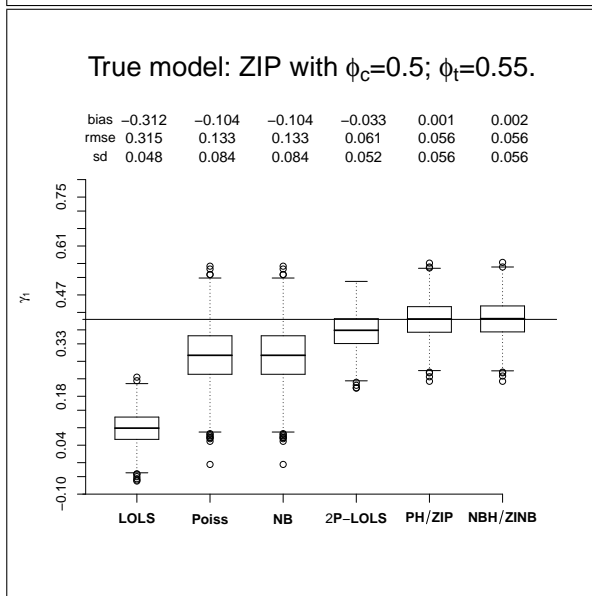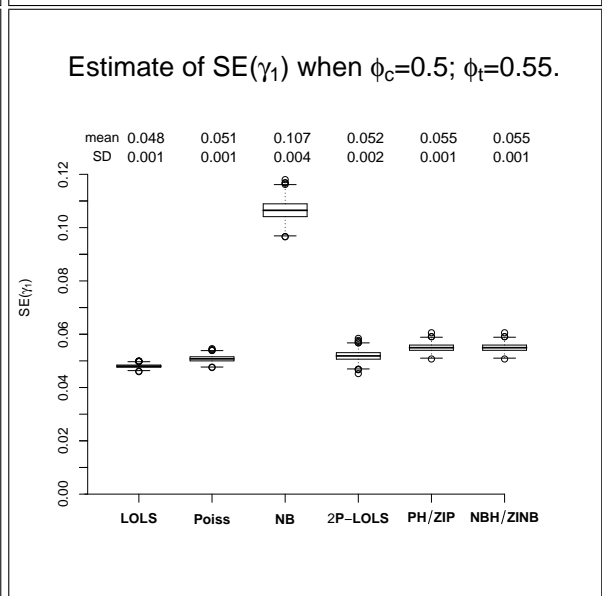

Supplement: S1 Fig — The box-plot of γ 1 estimates (in the left column panel) and their corresponding SE estimates (in the right column panel) for 1000 replications of simulated ZIP data using LOLS, Poisson, NB, 2P-LOLS and ZINB methods. The horizontal line in the left column plots is the true value of γ 1, which is 0.4. The consonant, neutral and dissonant scenarios are displayed in the first, second and third rows, respectively. The bias, root mean square error (rmse) and standard deviation (sd) of the estimations of γ 1 are shown above its box-plot for each method in the left column. The mean and standard deviation (sd) of the standard error (SE) estimations above the box-plot for each method in the right column. (PDF) [file pone.0129606.s013.pdf]

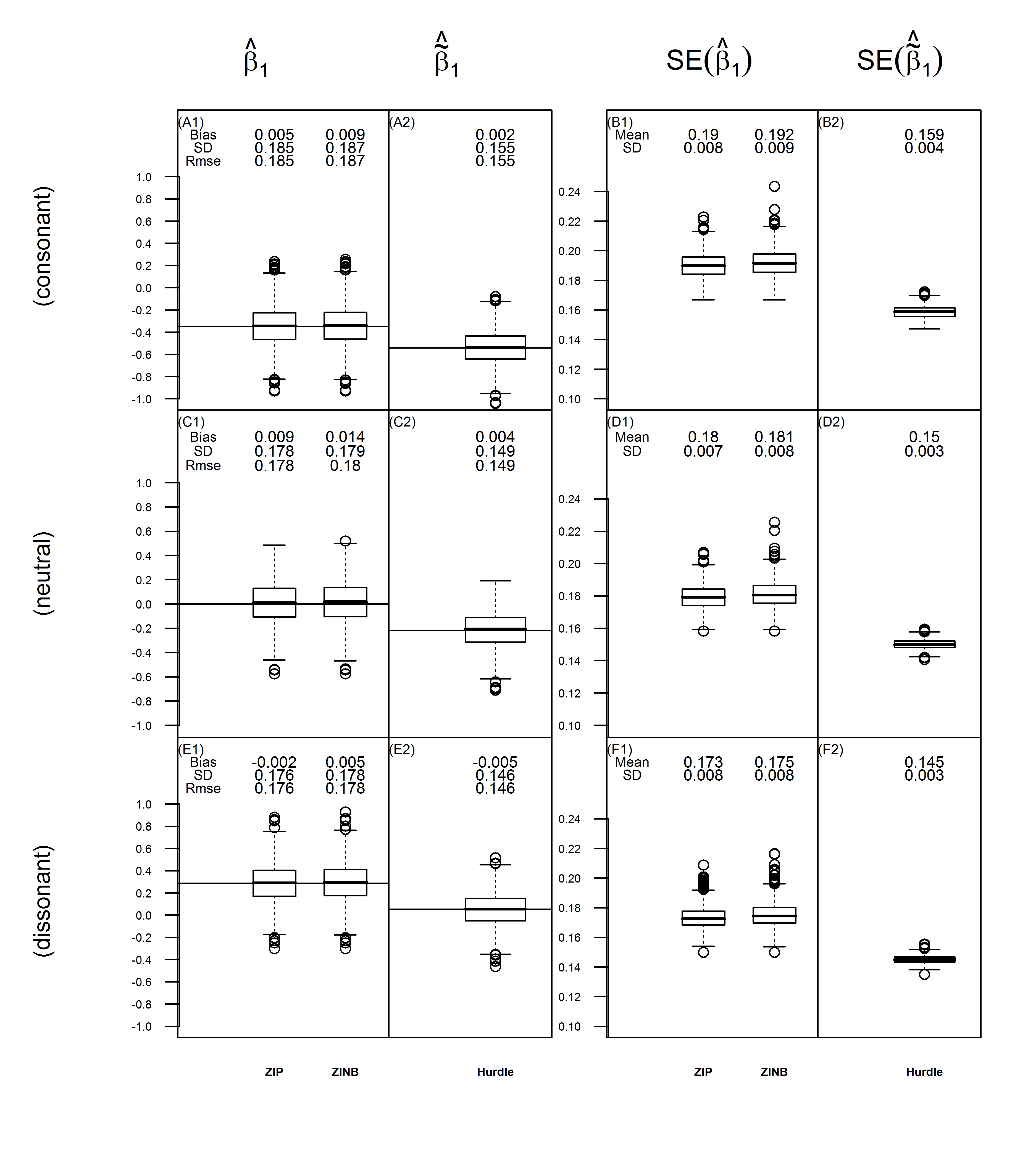

Supplement: S4 Fig — The figure displays box-plots of estimates and their standard errors for the covariate effect on the log-odds of structural zeroes for ZIP and ZINB method and on the log-odds of zeroes for hurdle models from 1000 replications. Panels (A1), (C1), and (E1) show the estimates of β 1 for consonant, neutral and dissonant effect case, respectively. The horizontal line in these panels represents the true value of β 1, which is −0.349 in (A1), 0 in (C1) and 0.287 in (E1). Panels (A2), (C2), and (E2) show the estimates of β˜1 for consonant, neutral and dissonant effect case, respectively. The horizontal line in these panels represents the true value of β˜1, which is −0.540 in (A2), −0.218 in (C2) and 0.053 in (E2). The bias, root mean square error (RMSE) and standard deviation (SD) of the estimates are shown above the box-plot for each method. Panel (B1), (D1), and (F1) show the SEs of the estimates for β 1, and panel (B2), (D2), and (F2) show the SEs of the estimates for β˜1. The mean and standard deviation (SD) of the standard error (SE) estimations are shown above the box-plot for each method. (TIFF) [file pone.0129606.s016.tiff]

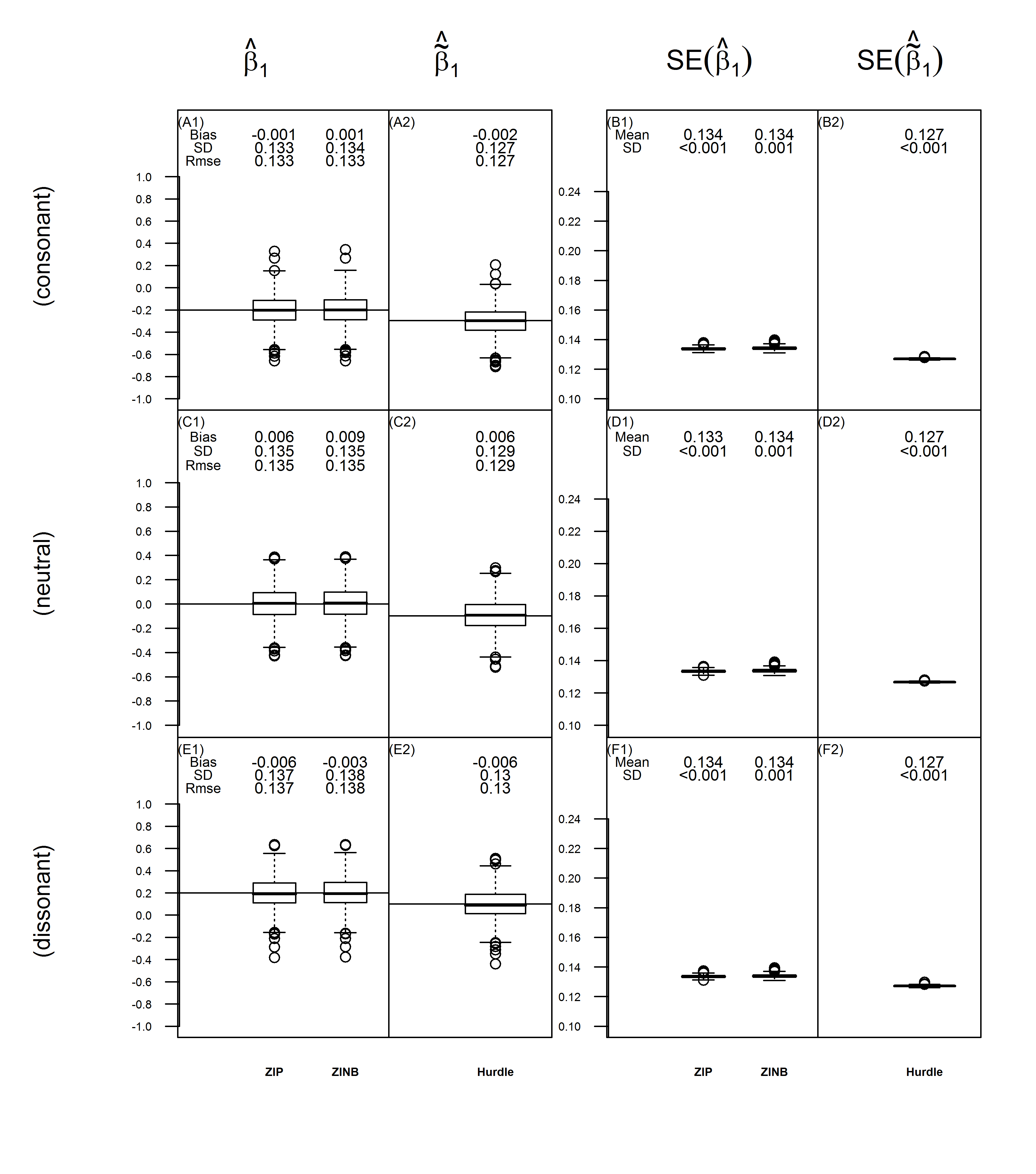

Supplement: S5 Fig — The figure displays box-plots of estimates and their standard errors for the covariate effect on the log-odds of structural zeroes for ZIP and ZINB method and on the log-odds of zeroes for hurdle models from 1000 replications. Panels (A1), (C1), and (E1) show the estimates of β 1 for consonant, neutral and dissonant effect case, respectively. The horizontal line in these panels represents the true value of β 1, which is −0.201 in (A1), 0 in (C1) and 0.201 in (E1). Panels (A2), (C2), and (E2) show the estimates of β˜1 for consonant, neutral and dissonant effect case, respectively. The horizontal line in these panels represents the true value of β˜1, which is −0.295 in (A2), −0.098 in (C2) and 0.100 in (E2). The bias, standard deviation (SD), and root mean square error (RMSE) of the estimates are shown above the box-plot for each method. Panel (B1), (D1), and (F1) show the SEs of the estimates for β 1, and panel (B2), (D2), and (F2) show the SEs of the estimates for β˜1. The mean and standard deviation (SD) of the standard error (SE) estimations are shown above the box-plot for each method. (TIFF) [file pone.0129606.s017.tiff]

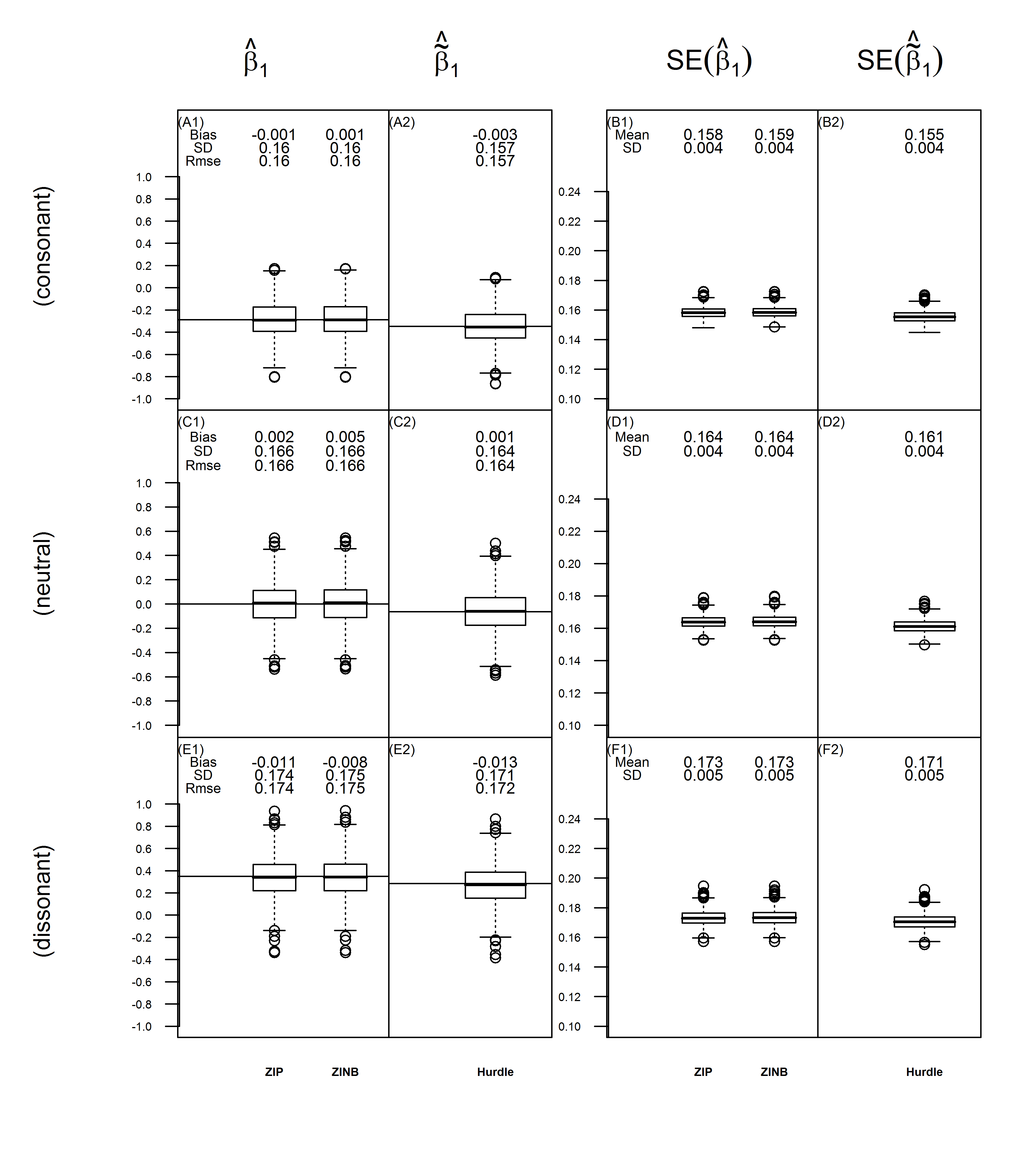

Supplement: S6 Fig — The figure displays box-plots of estimates and their standard errors for the covariate effect on the log-odds of structural zeroes for ZIP and ZINB method and on the log-odds of zeroes for hurdle models from 1000 replications. Panels (A1), (C1), and (E1) show the estimates of β 1 for consonant, neutral and dissonant effect case, respectively. The horizontal line in these panels represents the true value of β 1, which is −0.287 in (A1), 0 in (C1) and 0.349 in (E1). Panels (A2), (C2), and (E2) show the estimates of β˜1 for consonant, neutral and dissonant effect case, respectively. The horizontal line in these panels represents the true value of β˜1, which is −0.349 in (A2), −0.063 in (C2) and 0.285 in (E2). The bias, standard deviation (SD), and root mean square error (RMSE) of the estimates are shown above the box-plot for each method. Panel (B1), (D1), and (F1) show the SEs of the estimates for β 1, and panel (B2), (D2), and (F2) show the SEs of the estimates for β˜1. The mean and standard deviation (SD) of the standard error (SE) estimations are shown above the box-plot for each method. (TIFF) [file pone.0129606.s018.tiff]

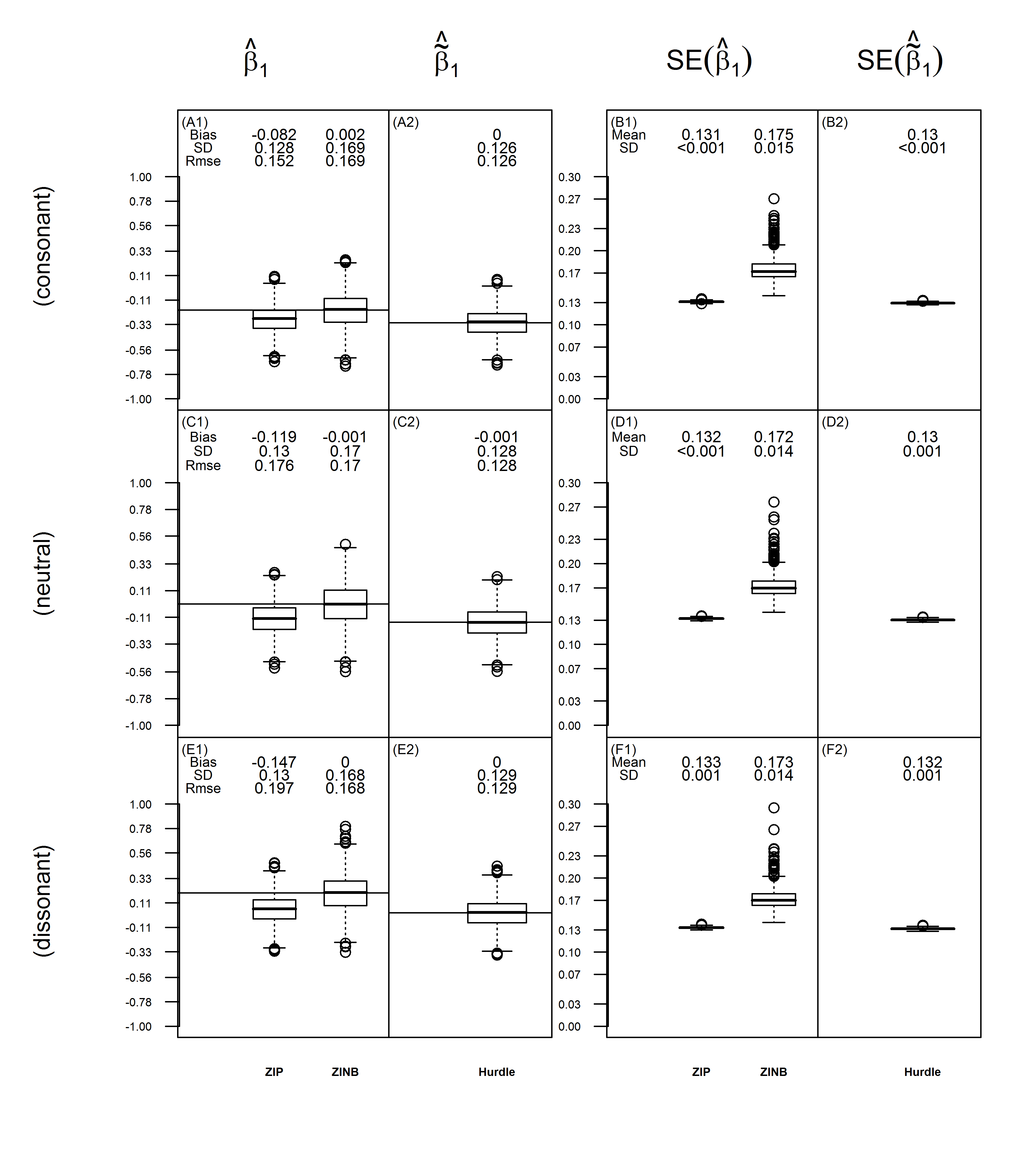

Supplement: S7 Fig — The figure displays box-plots of estimates and their standard errors for the covariate effect on the log-odds of structural zeroes for ZIP and ZINB method and on the log-odds of zeroes for hurdle models from 1000 replications. Panels (A1), (C1), and (E1) show the estimates of β 1 for consonant, neutral and dissonant effect case, respectively. The horizontal line in these panels represents the true value of β 1, which is −0.201 in (A1), 0 in (C1) and 0.201 in (E1). Panels (A2), (C2), and (E2) show the estimates of β˜1 for consonant, neutral and dissonant effect case, respectively. The horizontal line in these panels represents the true value of β˜1, which is −0.315 in (A2), −0.151 in (C2) and 0.020 in (E2). The bias, standard deviation (SD), and root mean square error (RMSE) of the estimates are shown above the box-plot for each method. Panel (B1), (D1), and (F1) show the SEs of the estimates for β 1, and panel (B2), (D2), and (F2) show the SEs of the estimates for β˜1. The mean and standard deviation (SD) of the standard error (SE) estimations are shown above the box-plot for each method. (TIFF) [file pone.0129606.s019.tiff]

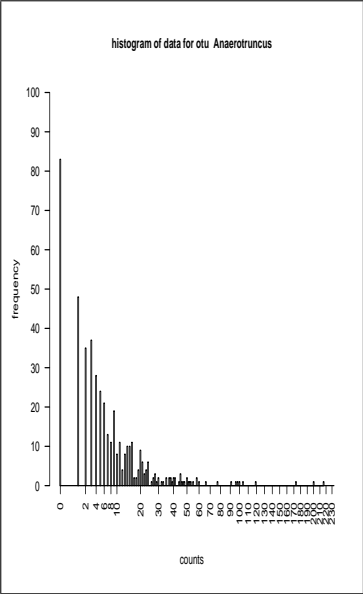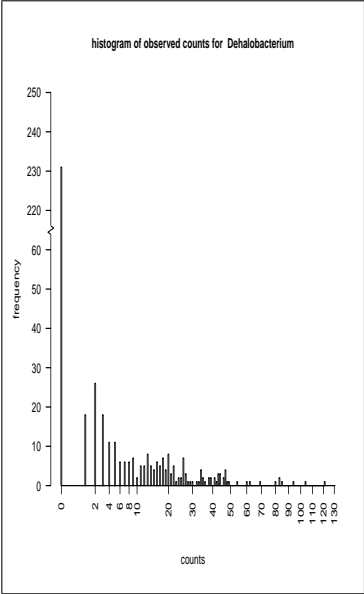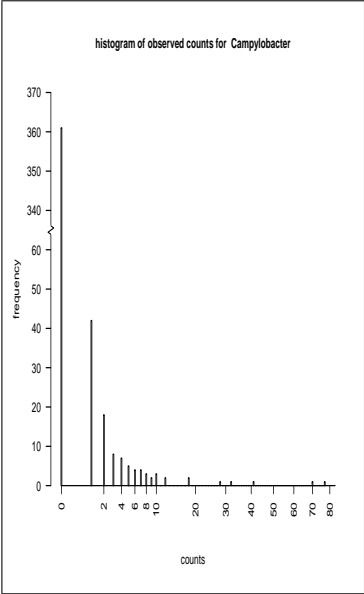

Supplement: S8 Fig — The X-axis is the possible counts of the bacterium in the square root scale. The Y-axis is the frequency of the counts with some line breaks. (PDF) [file pone.0129606.s020.pdf]

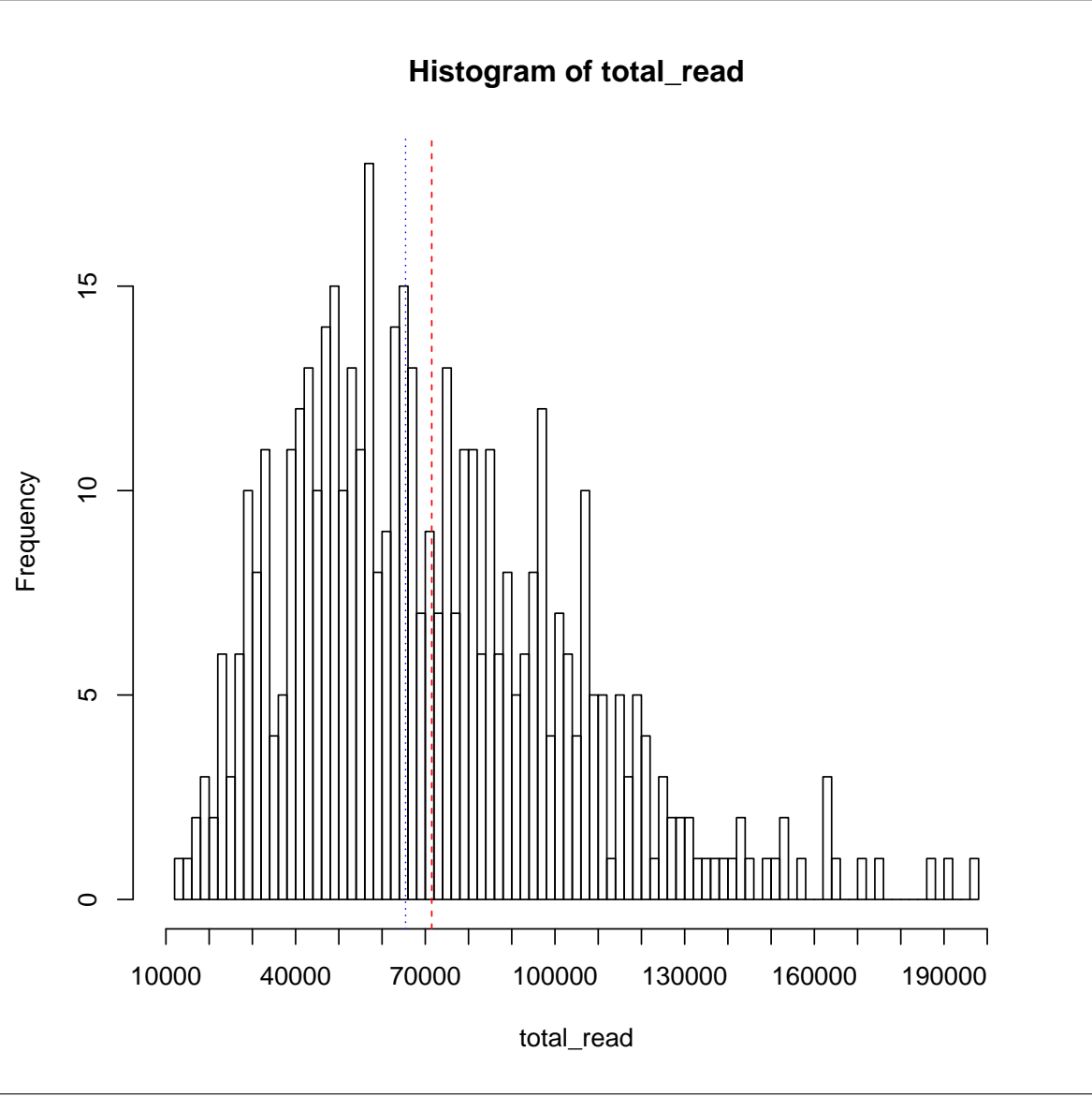

Supplement: S9 Fig — The red dashed line represent the mean of the total counts (71,490) and the blue dotted line represent the median of the total counts (65,438). The range is from 13,647 to 196,591. The standard deviation of the total counts is 32,839. (PDF) [file pone.0129606.s021.pdf]

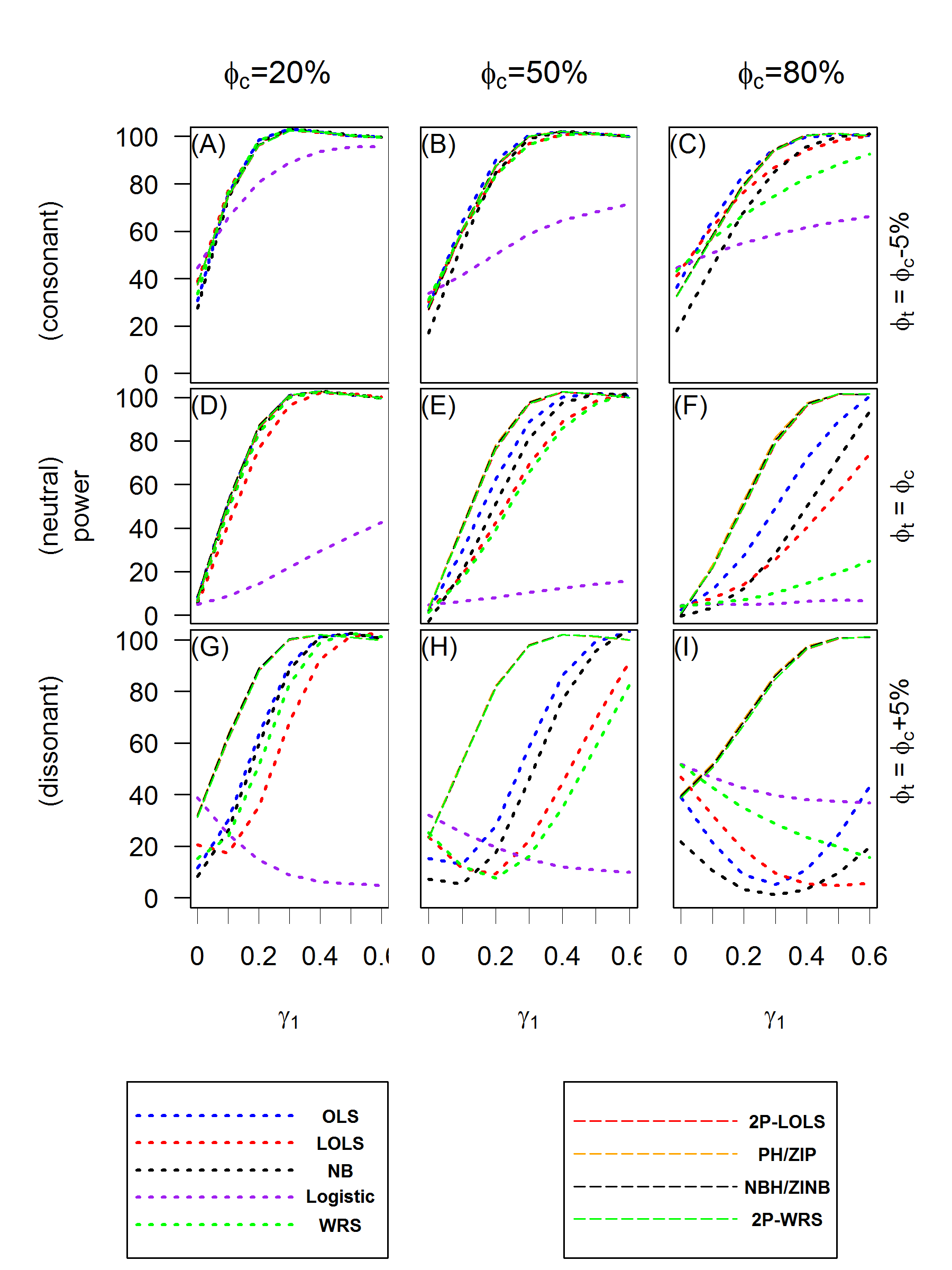

Supplement: S10 Fig — The X axis is the value of the covariate effect on the count data γ 1 and the Y axis is the power of test when the level of significance is 0.05. Three different cases of covariate effect, i.e., the consonant (ϕ t = ϕ c − 5%), neutral (ϕ t = ϕ c) and dissonant (ϕ t = ϕ c + 5%) effect, are presented in {(A), (B), (C)}, {(D), (E), (F)}, and {(G), (H), (I)}, respectively. Each column reflects different proportion of zero inflation in the non-exposed group: 20% in {(A), (D), (G)}, 50% in {(B), (E), (H)} and 80% in {(C), (F), (I)} from the first to the third column. (TIFF) [file pone.0129606.s022.tiff]

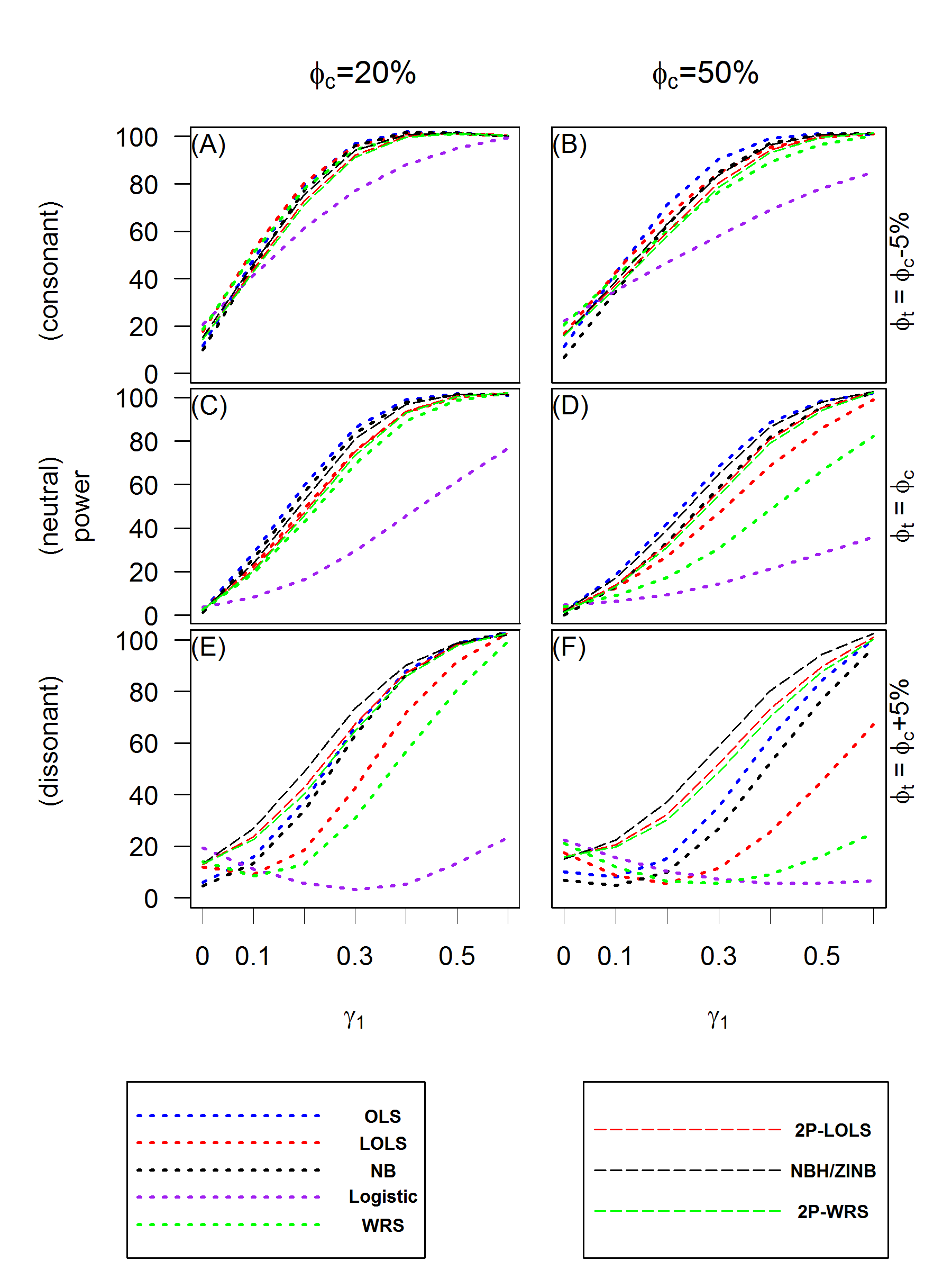

Supplement: S11 Fig — The X axis is the value of the covariate effect on the count data γ 1 and the Y axis is the power of test when the level of significance is 0.05. Three different cases of covariate effect, i.e., the consonant (ϕ t = ϕ c − 5%), neutral (ϕ t = ϕ c) and dissonant (ϕ t = ϕ c + 5%) effect, are presented in {(A), (B)}, {(C), (D)}, and {(E), (F)}, respectively. Each column reflects different proportion of zero inflation in the non-exposed group: 20% in {(A), (C), (E)} and 50% in {(B), (D), (F)} from the left to the right column, respectively. (TIFF) [file pone.0129606.s023.tiff]
