# Supplementary material for: Assessment and Selection of Competing Models for Zero-Inflated Microbiome Data
Source: PLoS One. 2015 Jul 6;10(7):e0129606. doi: 10.1371/journal.pone.0129606 (PMC4493133; doi:10.1371/journal.pone.0129606)
Supplement: S2 Fig — The box-plot of γ 1 estimates (in the left column panel) and their corresponding SE estimates (in the right column panel) for 1000 replications of simulated ZIP data using LOLS, Poisson, NB, 2P-LOLS and ZINB methods. The horizontal line in the left column plots is the true value of γ 1, which is 0.4. The consonant, neutral and dissonant scenarios are displayed in the first, second and third rows, respectively. The bias, root mean square error (rmse) and standard deviation (sd) of the estimations of γ 1 are shown above its box-plot for each method in the left column. The mean and standard deviation (sd) of the standard error (SE) estimations above the box-plot for each method in the right column. (PDF) [file pone.0129606.s014.pdf]

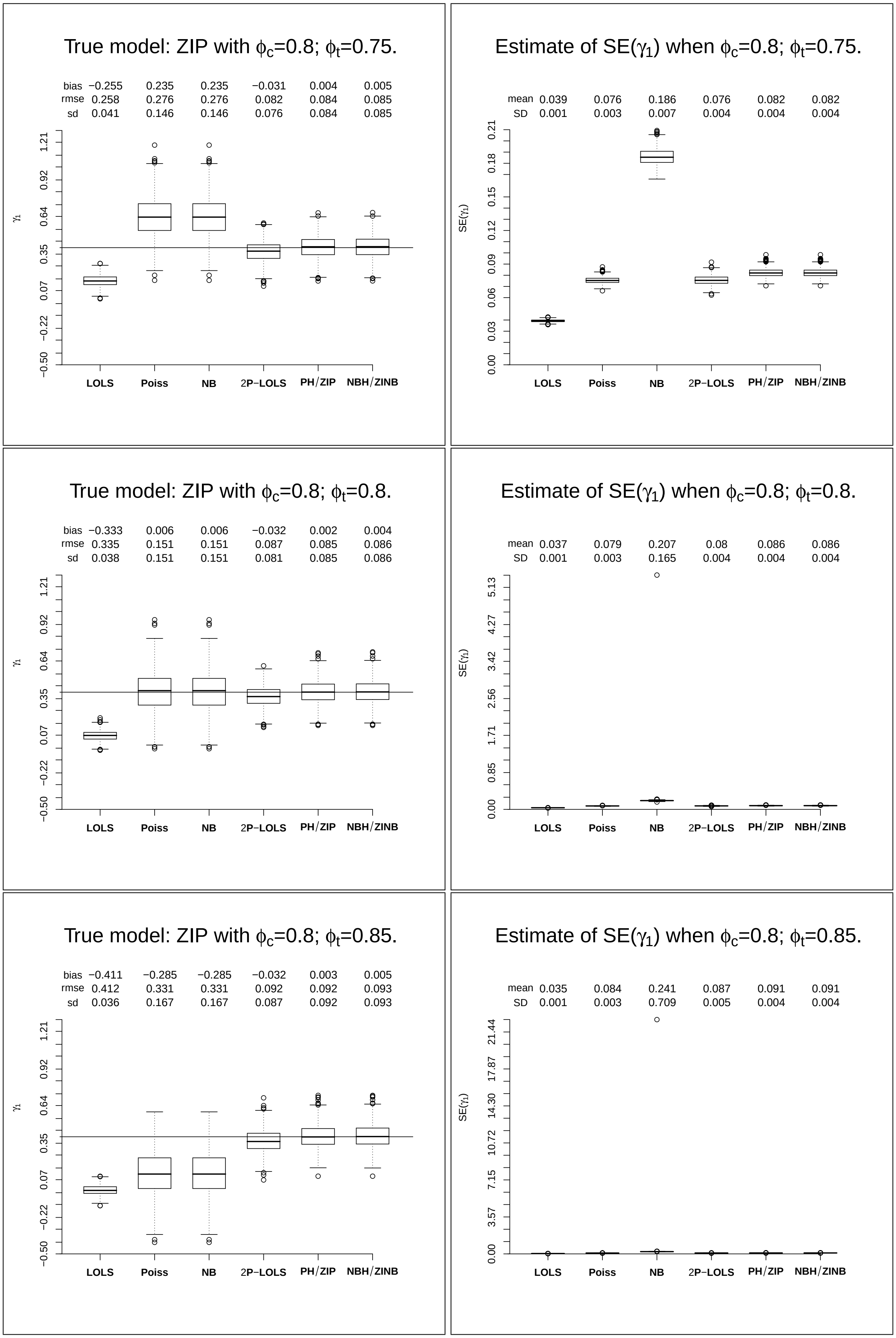
True model: ZIP with ϕc=0.8; ϕt=0.75.
bias −0.255 0.235 0.235 −0.031 0.004 0.005
rmse 0.258 0.276 0.276 0.082 0.084 0.085
sd 0.041 0.146 0.146 0.076 0.084 0.085
γ1
LOLS Poiss NB 2P−LOLS PH/ZIP NBH/ZINB
Estimate of SE(γ1) when ϕc=0.8; ϕt=0.75.
mean 0.039 0.076 0.186 0.076 0.082 0.082
SD 0.001 0.003 0.007 0.004 0.004 0.004
SE(γ1)
LOLS Poiss NB 2P−LOLS PH/ZIP NBH/ZINB
True model: ZIP with ϕc=0.8; ϕt=0.8.
bias −0.333 0.006 0.006 −0.032 0.002 0.004
rmse 0.335 0.151 0.151 0.087 0.085 0.086
sd 0.038 0.151 0.151 0.081 0.085 0.086
γ1
LOLS Poiss NB 2P−LOLS PH/ZIP NBH/ZINB
Estimate of SE(γ1) when ϕc=0.8; ϕt=0.8.
mean 0.037 0.079 0.207 0.08 0.086 0.086
SD 0.001 0.003 0.165 0.004 0.004 0.004
SE(γ1)
LOLS Poiss NB 2P−LOLS PH/ZIP NBH/ZINB
True model: ZIP with ϕc=0.8; ϕt=0.85.
bias −0.411 −0.285 −0.285 −0.032 0.003 0.005
rmse 0.412 0.331 0.331 0.092 0.092 0.093
sd 0.036 0.167 0.167 0.087 0.092 0.093
γ1
LOLS Poiss NB 2P−LOLS PH/ZIP NBH/ZINB
Estimate of SE(γ1) when ϕc=0.8; ϕt=0.85.
mean 0.035 0.084 0.241 0.087 0.091 0.091
SD 0.001 0.003 0.709 0.005 0.004 0.004
SE(γ1)
LOLS Poiss NB 2P−LOLS PH/ZIP NBH/ZINB
