# Supplementary material for: Assessment and Selection of Competing Models for Zero-Inflated Microbiome Data
Source: PLoS One. 2015 Jul 6;10(7):e0129606. doi: 10.1371/journal.pone.0129606 (PMC4493133; doi:10.1371/journal.pone.0129606)
Supplement: S3 Fig — The box-plot of γ 1 estimates (in the left column panel) and their corresponding SE estimates (in the right column panel) for 1000 replications of simulated ZINB data using LOLS, Poisson, NB, 2P-LOLS and ZINB methods. The horizontal line in the left column plots is the true value of γ 1, which is 0.4. The consonant, neutral and dissonant scenarios are displayed in the first, second and third rows, respectively. The bias, root mean square error (rmse) and standard deviation (sd) of the estimations of γ 1 are shown above its box-plot for each method in the left column. The mean and standard deviation (sd) of the standard error (SE) estimations above the box-plot for each method in the right column. (PDF) [file pone.0129606.s015.pdf]

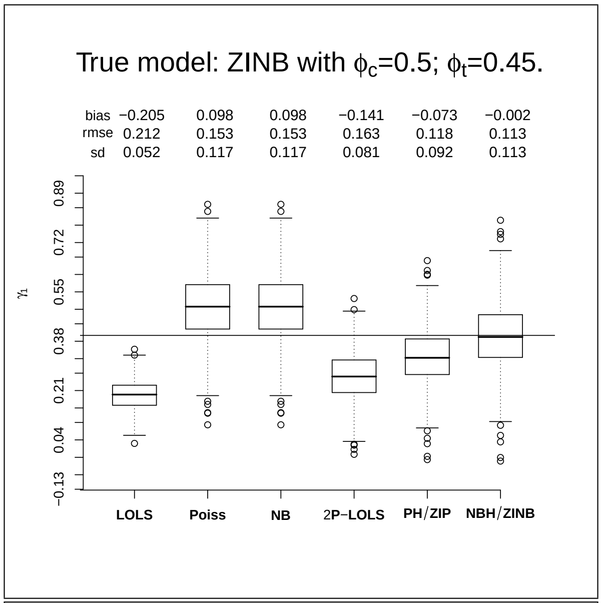
True model: ZINB with ϕc=0.5; ϕt=0.45.
bias −0.205 0.098 0.098 −0.141 −0.073 −0.002
rmse 0.212 0.153 0.153 0.163 0.118 0.113
sd 0.052 0.117 0.117 0.081 0.092 0.113
γ1
−0.13 0.04 0.21 0.38 0.55 0.72 0.89
LOLS Poiss NB 2P−LOLS PH/ZIP NBH/ZINB

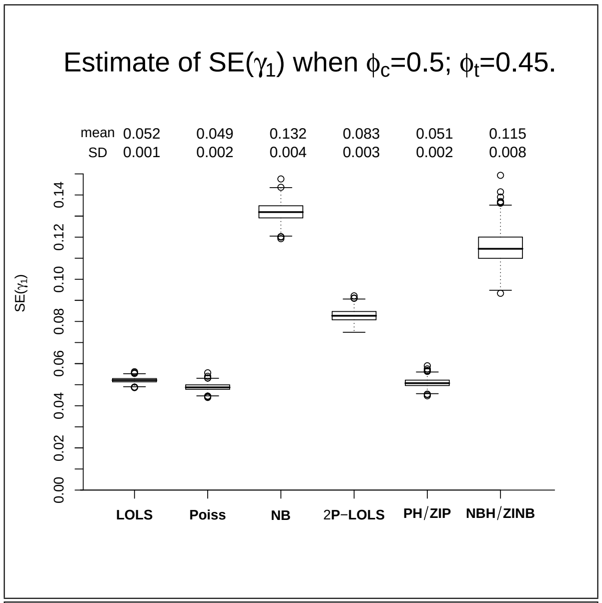
Estimate of SE(γ1) when ϕc=0.5; ϕt=0.45.
mean 0.052 0.049 0.132 0.083 0.051 0.115
SD 0.001 0.002 0.004 0.003 0.002 0.008
SE(γ1)
0.00 0.02 0.04 0.06 0.08 0.10 0.12 0.14
LOLS Poiss NB 2P−LOLS PH/ZIP NBH/ZINB

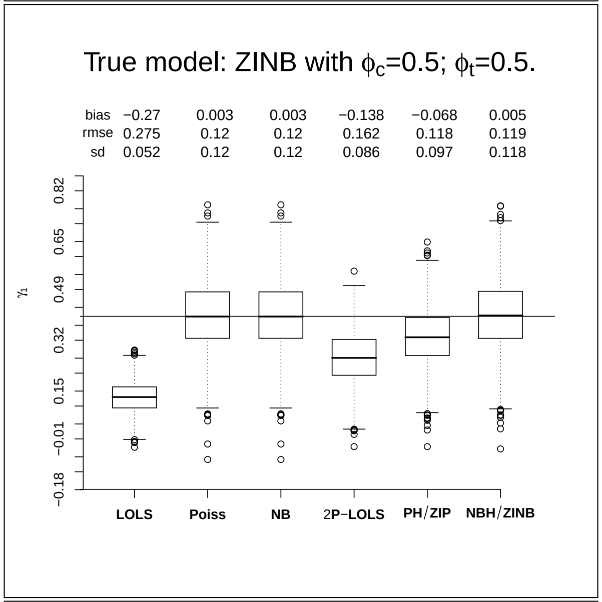
True model: ZINB with ϕc=0.5; ϕt=0.5.
bias −0.27 0.003 0.003 −0.138 −0.068 0.005
rmse 0.275 0.12 0.12 0.162 0.118 0.119
sd 0.052 0.12 0.12 0.086 0.097 0.118
γ1
−0.18 −0.01 0.15 0.32 0.49 0.65 0.82
LOLS Poiss NB 2P−LOLS PH/ZIP NBH/ZINB

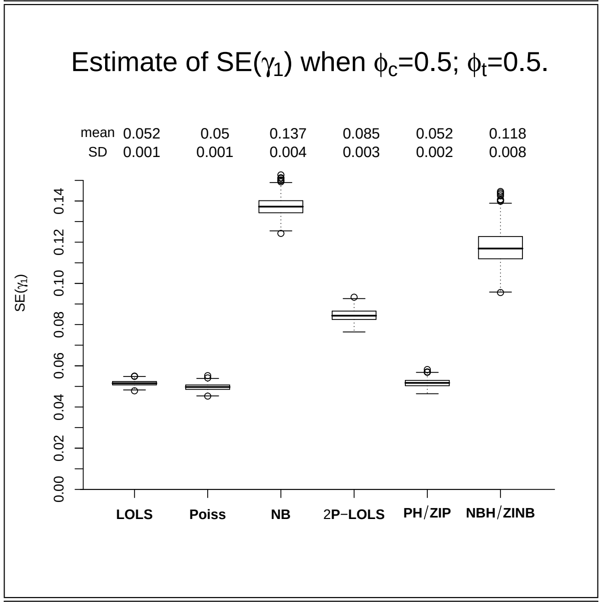
Estimate of SE(γ1) when ϕc=0.5; ϕt=0.5.
mean 0.052 0.05 0.137 0.085 0.052 0.118
SD 0.001 0.001 0.004 0.003 0.002 0.008
SE(γ1)
0.00 0.02 0.04 0.06 0.08 0.10 0.12 0.14
LOLS Poiss NB 2P−LOLS PH/ZIP NBH/ZINB

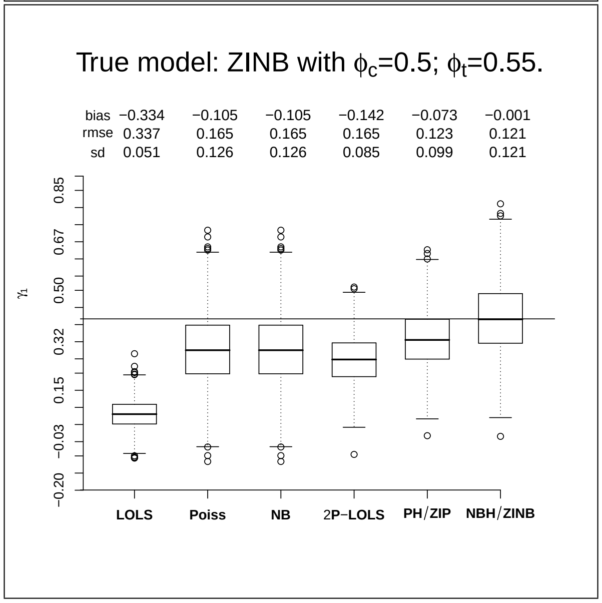
True model: ZINB with ϕc=0.5; ϕt=0.55.
bias −0.334 −0.105 −0.105 −0.142 −0.073 −0.001
rmse 0.337 0.165 0.165 0.165 0.123 0.121
sd 0.051 0.126 0.126 0.085 0.099 0.121
γ1
−0.20 −0.03 0.15 0.32 0.50 0.67 0.85
LOLS Poiss NB 2P−LOLS PH/ZIP NBH/ZINB

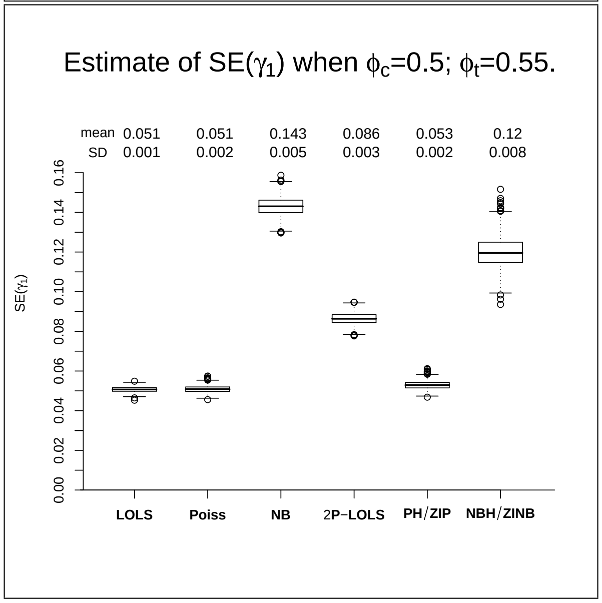
Estimate of SE(γ1) when ϕc=0.5; ϕt=0.55.
mean 0.051 0.051 0.143 0.086 0.053 0.12
SD 0.001 0.002 0.005 0.003 0.002 0.008
SE(γ1)
0.00 0.02 0.04 0.06 0.08 0.10 0.12 0.14 0.16
LOLS Poiss NB 2P−LOLS PH/ZIP NBH/ZINB
